# Bipyridine complexes of E^3+^ (E = P, As, Sb, Bi): strong Lewis acids, sources of E(OTf)_3_ and synthons for E^I^ and E^V^ cations†
†Electronic supplementary information (ESI) available. CCDC 1410568–1410574. For ESI and crystallographic data in CIF or other electronic format see DOI: 10.1039/c5sc02423d


**DOI:** 10.1039/c5sc02423d

**Published:** 2015-08-03

**Authors:** Saurabh S. Chitnis, Alasdair P. M. Robertson, Neil Burford, Brian O. Patrick, Robert McDonald, Michael J. Ferguson

**Affiliations:** a Department of Chemistry , University of Victoria , Victoria , British Columbia V8W 3V6 , Canada . Email: nburford@uvic.ca ; Fax: +1 250 721 7147 ; Tel: +1 250 721 7150; b Department of Chemistry , University of British Columbia , Vancouver , British Columbia V6T 1Z1 , Canada; c Department of Chemistry , University of Alberta , Edmonton , Alberta T6G 2T2 , Canada

## Abstract

Triflate salts of trications [(bipy)_2_E]^3+^ ([**6E**][OTf]_3_) and [(tbbipy)_2_E]^3+^ ([**6′E**][OTf]_3_) (bipy = 2,2′-bipyridine, tbbipy = 4,4′-di-^*t*^butyl-2,2′-bipyridine; E = P, As, Sb, Bi) have been synthesized and comprehensively characterized. The unique molecular and electronic structures of this new class of complexes involving pnictogen Lewis acids has been assessed in the solid, solution and gas phases to reveal systematic variations in metric parameters, ligand lability and charge concentration. While the Lewis acidity of E^3+^ has the trend E = Bi < Sb < As < P as determined by gas-phase calculations and ^1^H NMR spectroscopy, the Lewis acidity of [**6E**]^3+^ has the trend E = P < As < Sb < Bi according to gas-phase calculations. Derivatives of [**6′E**][OTf]_3_ (E = P, As) are latent sources of E(OTf)_3_ as demonstrated by their reactions with dmap, which give the corresponding derivatives of [(dmap)_3_E][OTf]_3_. The highly oxidizing nature of P(OTf)_3_ and As(OTf)_3_ is evidenced in reactions of [**6′E**][OTf]_3_ (E = P, As) with phosphines, which give E^I^-containing monocations [(R_3_P)_2_E]^1+^ and oxidatively coupled dications [R_3_PPR_3_]^2+^, illustrating new P–P and P–As bond forming strategies. Cations [**6′E**]^3+^ (E = P, As) are C–H bond activating agents that dehydrogenate 1,4-cyclohexadiene, with higher activity observed for E = P. Combinations of [**6′E**]^3+^ and ^*t*^Bu_3_P activate H_2_ and D_2_ under mild conditions, evidencing frustrated Lewis pair activity. Oxidation of [**6′P**][OTf]_3_ with SO_2_Cl_2_ gives [(tbbipy)_2_PCl_2_][OTf]_3_, containing a P^V^-trication, but there is no evidence of the analogous reaction with [**6′As**][OTf]_3_. The observations highlight new directions in the chemistry of highly charged cations and reveal a rich reactivity for p-block triflates E(OTf)_3_, which can be accessed through derivatives of [**6E**][OTf]_3_ and [**6′E**][OTf]_3_.

## Abstract 2

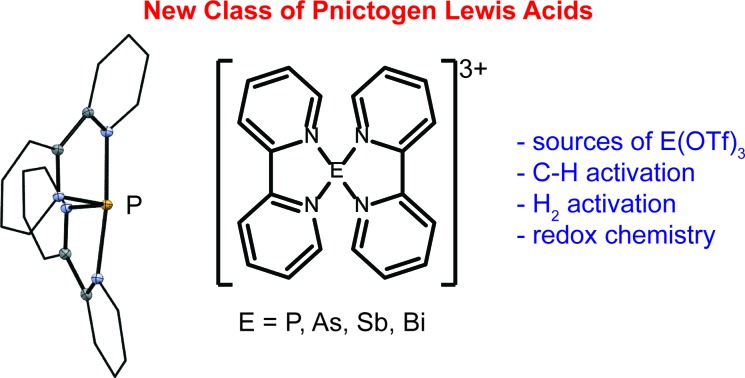
Triflate salts of trications [(bipy)_2_E]^3+^ ([**6E**][OTf]_3_) and [(tbbipy)_2_E]^3+^ ([**6′E**][OTf]_3_) (bipy = 2,2′-bipyridine, tbbipy = 4,4′-di-^*t*^butyl-2,2′-bipyridine; E = P, As, Sb, Bi) have been synthesized and comprehensively characterized.

## Introduction

**Chart 1:**
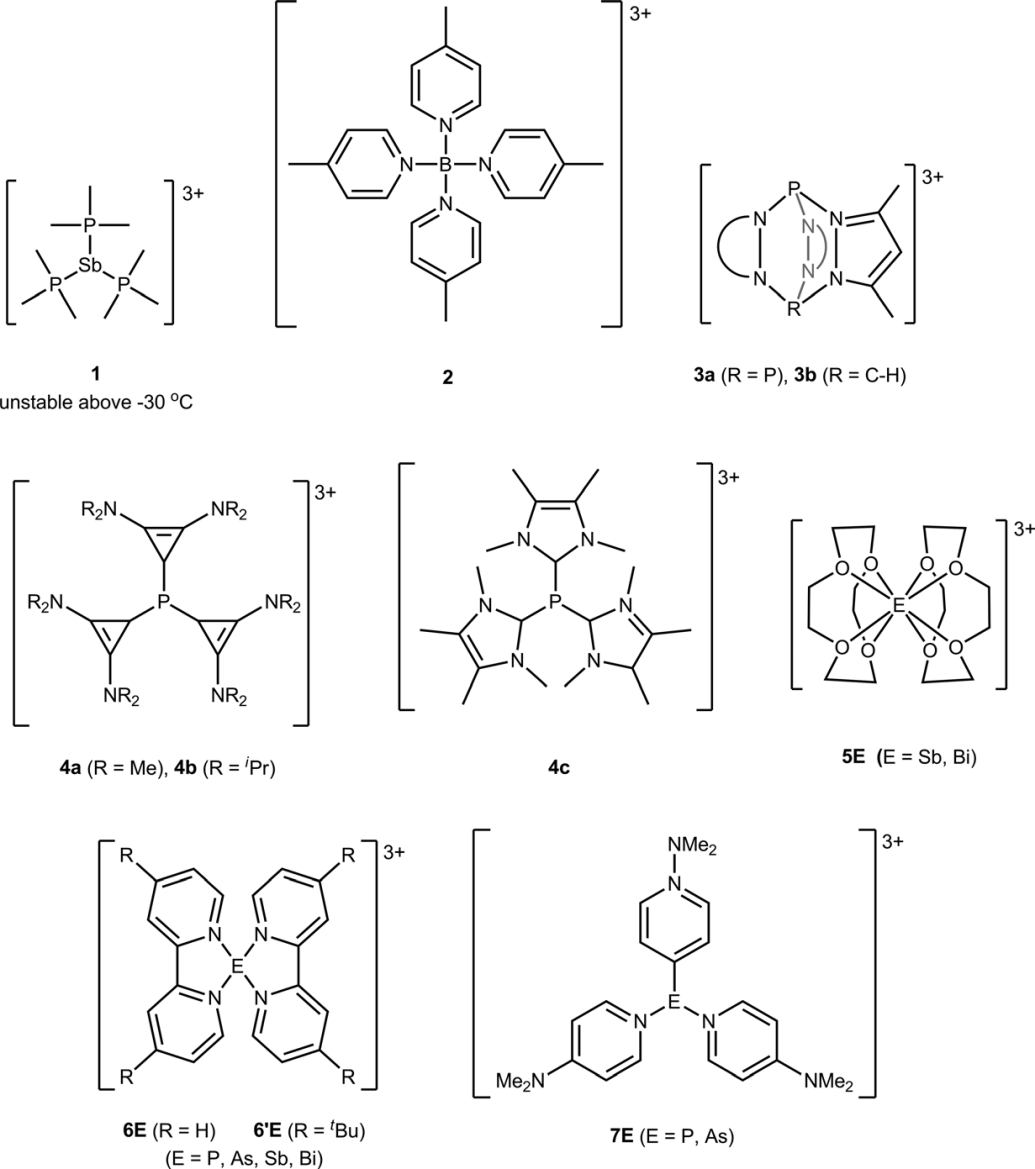
p-Block element centered tricationic complexes.

Numerous monocationic and dicationic p-block element centered complexes are known,^1^ but structurally authenticated salts containing trications are rare, because the charge concentration often results in oxidation of the ligands. For example, the trisphosphine-antimony trication **1** (Chart 1) undergoes reductive elimination of a diphosphonium dication below room temperature.^2^,^3^ In this context, the pyridine ligands in **2**,^4^ the tris-pyrazole based ligands in **3a,b**,^5^ the carbene based ligands in **4a–c**,^6^ and the crown ether ligands in **5E**^7^ illustrate types of oxidatively resistant donors that enable studies of such reactive coordination centers.

As a prototypical ligand for transition metal acceptors in a variety of oxidation states, 2,2′-bipyridine (bipy) offers relatively high basicity and oxidative resistance, which we have now exploited to enable a comprehensive study of a series of compounds of generic formulae [(bipy)_2_E][OTf]_3_, [**6E**][OTf]_3_, and [(tbbipy)_2_E][OTf]_3_, [**6′E**][OTf]_3_ (E = P, As, Sb, Bi; tbbipy = 4,4′-di-^*t*^butyl-2,2′-bipyridine).^8^ The compounds are characterized as salts containing trications that represent bipy or tbbipy complexes of E^3+^. A diverse reactivity is evident for these complexes, including ligand exchange, which provides access to the dmap complexes [**7E**][OTf]_3_ (E = P, As). Element triflates, E(OTf)_*n*_, are widely employed as Lewis acids,^9^ oxidizing agents^10^–^12^ and latent sources of E^*n*+^.^13^–^15^ Interesting examples of small-molecule activation and catalysis effected by p-block element triflate salts are also well documented,^16^–^18^ including those involving Sb(OTf)_3_ 19 and Bi(OTf)_3_,^20^ which can be isolated on preparative scales,^21^,^22^ enabling their ubiquitous use. Lighter congeners featuring the more electronegative P and As centers have not yet been reported, precluding assessment of their reactivity. In this context, we demonstrate that derivatives of [**6E**][OTf]_3_ and [**6′E**][OTf]_3_ represent examples of E(OTf)_3_ transfer reagents, C–H and H–H bond activating reagents, and synthons for E^I^- and E^V^-centered cations.

## Results and discussion

**Scheme 1:**
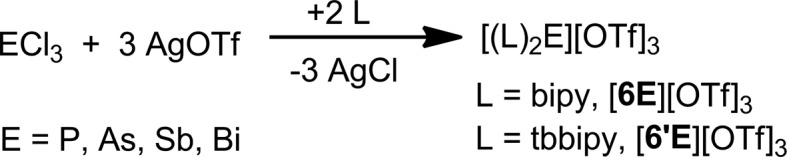
Synthesis of [**6E**][OTf]_3_ and [**6′E**][OTf]_3_.

Complexes of E(OTf)_3_ with bipy or tbbipy were prepared according to Scheme 1 and isolated as crystalline solids. While all derivatives decompose to give the protonated ligand on exposure to ambient atmosphere, they can be stored indefinitely under inert atmosphere at room temperature. Derivatives of [**6E**][OTf]_3_ are less soluble in MeCN and CH_2_Cl_2_ than derivatives of [**6′E**][OTf]_3_, due to the presence of four ^*t*^butyl groups in the latter. Interestingly, while the phosphorus derivatives are yellow due to a HOMO–LUMO transition centered around 300 nm,^23^ all other derivatives are colourless as solids or in MeCN solutions.

**Fig. 1:**
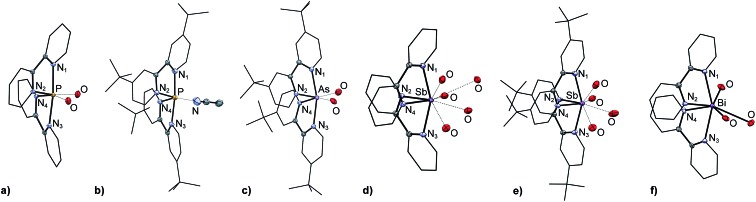
Solid-state molecular structures of the cations in (a) [**6P**][OTf]_3_·2MeCN, (b) [**6′P**][OTf]_3_·MeCN, (c) [**6′As**][OTf]_3_·2.83MeCN, (d) [**6Sb**][OTf]_3_·MeCN, (e) [**6′Sb**][OTf]_3_·MeCN, and (f) [**6Bi**][OTf]_3_. Hydrogen atoms, non-interacting portions of the triflate anions and solvent molecules have been omitted for clarity.

The solid-state structures of [**6P**][OTf]_3_·2MeCN, [**6′P**][OTf]_3_·MeCN, [**6′As**][OTf]_3_·2.83MeCN, [**6Sb**][OTf]_3_·MeCN, [**6′Sb**][OTf]_3_·MeCN, and [**6Bi**][OTf]_3_·MeCN have been determined to reveal spirocyclic environments for E with four E–N bonds (10-E-4 as per the Arduengo nomenclature^24^) and varying degrees of E–O triflate contacts, as shown in Fig. 1. Selected metric parameters for derivatives of [**6E**][OTf]_3_ and [**6′E**][OTf]_3_ are collated in Table 1, where computationally determined (gas phase) values are listed for [**6As**]^3+^ and [**6′Bi**]^3+^, for which experimental solid state data are not available. In all cases, the structures indicate the stereochemical presence of a lone pair at the acceptor pnictogen centre. The intermolecular MeCN···P interaction for [**6′P**][OTf]_3_·MeCN (coordinated solvent) and interion O···E interactions (triflate anions) in [**6P**][OTf]_3_, [**6′P**][OTf]_3_·MeCN, [**6′As**][OTf]_3_·2.83MeCN, and [**6′Sb**][OTf]_3_·MeCN are closer in magnitude to ∑_r,vdW_ than to ∑_r,cov_ for the elements involved. In contrast, short Bi···O interactions are observed in [**6Bi**][OTf]_3_, representing elongated Bi–O covalent bonds rather than triflate anions interacting with a bismuth cation. We therefore classify all derivatives as ionic except [**6Bi**][OTf]_3_ which is best described in the solid state as a bis-bipy adduct of Bi(OTf)_3_.

**Table 1:** Selected bond lengths (Å) and angles (°) in the solid-state structures of [**6P**][OTf]_3_·2MeCN, [**6′P**][OTf]_3_·MeCN, [**6′As**][OTf]_3_·2.83MeCN, [**6Sb**][OTf]_3_·MeCN,^8^ [**6′Sb**][OTf]_3_·MeCN, and [**6Bi**][OTf]_3_, calculated (gas phase, PBE0/def2-TZVP) values for cations [**6As**]^3+^ and [**6′Bi**]^3+^, and sums of covalent (∑_r,cov_)^25^ and van der Waals (∑_r,vdW_)^26^,^27^ radii for selected atom pairs

	[**6P**][OTf]_3_ ·2MeCN	[**6′P**][OTf]_3_ ·MeCN	[**6As**]^3+^	[**6′As**][OTf]_3_ ·2.83MeCN	[**6Sb**][OTf]_3_ ·MeCN	[**6′Sb**][OTf]_3_ ·MeCN	[**6Bi**][OTf]_3_ ·MeCN	[**6′Bi**]^3+^
E–N_1_	1.939(2)	1.971(2)	2.0993	2.124(2)	2.284(2)	2.269(2)	2.454(6)	2.3461
E–N_2_	1.811(2)	1.812(2)	1.9714	1.997(2)	2.233(2)	2.198(2)	2.364(6)	2.2519
E–N_3_	1.974(2)	1.959(2)	2.0993	2.125(2)	2.332(2)	2.310(2)	2.430(6)	2.3461
E–N_4_	1.816(2)	1.811(2)	1.9714	1.999(2)	2.243(2)	2.218(2)	2.372(6)	2.2519
∑_r,cov_(E, N)	1.82	1.82	1.92	1.92	2.11	2.11	2.22	2.22
∑_r,vdW_(E, N)	3.35	3.35	3.40	3.40	3.61	3.61	3.62	3.62
E–O_OTf_	3.006(2)		—	2.705(2)	2.598(2)	2.586(2)	2.607(6)	—
	3.109(2)			2.742(2)	2.650(2)	3.113(2)	2.532(6)	
					3.077(1)	2.767(2)	2.893(6)	
					3.247(1)	3.343(2)		
					3.367(1)			
∑_r,cov_(E, O)	1.74	1.74	1.84	1.84	2.03	2.03	2.14	2.14
∑_r,vdW_(E, O)	3.32	3.32	3.37	3.37	3.58	3.58	3.59	3.59
N_1_–E–N_2_	82.20(7)	81.61(8)	78.86	77.67(8)	72.09(4)	72.43(5)	68.1(2)	71.53
N_3_–E–N_4_	82.07(7)	82.25(8)	78.86	77.76(8)	71.53(4)		68.2(2)	71.53
N_1_–E–N_3_	173.09(8)	173.10(8)	168.47	162.75(8)	156.02(4)	153.45(5)	154.4(2)	152.92
N_1_–E–N_4_	92.91(7)	92.63(8)	93.47	89.96(8)	87.87(4)	89.17(5)	94.1(2)	90.16
N_2_–E–N_3_	93.36(7)	94.44(8)	93.47	90.45(8)	91.42(4)	86.70(5)	88.8(2)	90.16
N_2_–E–N_4_	99.57(8)	97.56(8)	97.39	91.54(8)	78.66(4)	81.98(5)	74.7(2)	95.78

The bond angles for a given E in [**6E**][OTf]_3_ and [**6′E**][OTf]_3_ are expected to be very similar because the divergent planes defined by N_1_–E–N_2_ and N_3_–E–N_4_ result in the ^*t*^Bu groups facing away from each other, so that steric repulsion between them is minimal. For example, the quaternary carbon centers in the ^*t*^Bu groups that are *para* to N_2_ and N_4_ in [**6′P**][OTf]_3_ are separated by nearly 10 Å, and the bond angles within the disphenoidal frames of [**6P**]^3+^ and [**6′P**]^3+^ are essentially identical, as they are for [**6Sb**]^3+^ and [**6′Sb**]^3+^. The inductive effect of a ^*t*^Bu group *para* to the nitrogen atoms is expected to make the tbbipy ligands more basic compared to bipy and lead to stronger E–N interactions. Consistently, the ^31^P NMR spectrum of a CD_3_CN mixture containing tbbipy and [**6P**][OTf]_3_ in a 2 : 1 stoichiometry showed a broad peak corresponding to [**6P′**][OTf]_3_. The E–N distances in [**6P**][OTf]_3_·2MeCN and [**6′P**][OTf]_3_·MeCN are similar, although the presence of a coordinated MeCN donor in the latter may reduce the electrophilicity of the phosphorus center and offset the expected E–N shortening. Better suited for direct comparison are [**6Sb**][OTf]_3_·MeCN and [**6′Sb**][OTf]_3_·MeCN, where the MeCN molecule in the lattice does not interact with the Sb centers. Evidencing the inductive effect of the *para*-^*t*^Bu group, E–N distances in [**6′Sb**][OTf]_3_·MeCN are on average 0.1 Å shorter than the bipy derivative, which also shows two more Sb–O interion contacts than does the tbbipy derivative. Moreover, the Sb–O contacts in [**6′Sb**][OTf]_3_·MeCN are on average 0.05 Å longer than those in [**6Sb**][OTf]_3_·MeCN. These observations support a slightly greater Lewis basicity for tbbipy, which is discernible in the E–N distances, in the absence of additional donors (*e.g.* coordinated solvent) at E.

**Fig. 2:**
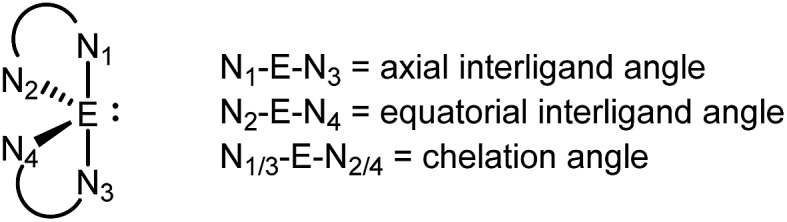
Definition of key angles in the disphenoidal geometry of [**6E**]^3+^.

The *trans* configured E–N bonds in all derivatives are *ca.* 0.1 Å longer than the E–N bonds in equatorial positions due to the mutual *trans* influence of the E–N_1_ and E–N_3_ interactions (Fig. 2). For the equatorial positions, the trend in E–N bond lengths, [**6P**]^3+^ ≈ [**6′P**]^3+^ < [**6′As**]^3+^ < [**6Sb**]^3+^ ≈ [**6′Sb**]^3+^ < [**6Bi**]^3+^, reflects the sum of the respective covalent radii (∑_r,cov_). The trend in the axial (N_1_–E–N_3_) and equatorial interligand angles (N_2_–E–N_4_), [**6P**]^3+^ ≈ [**6′P**]^3+^ > [**6′As**]^3+^ > [**6Sb**]^3+^ ≈ [**6′Sb**]^3+^ > [**6Bi**]^3+^, is consistent and is attributed to the extent of triflate anion association, which is greater for an atom with a larger atomic radius. The chelation angles (N_1/3_–E–N_2/4_) exhibit the trend [**6P**]^3+^ ≈ [**6′P**]^3+^ > [**6′As**]^3+^ > [**6Sb**]^3+^ ≈ [**6′Sb**]^3+^ > [**6Bi**]^3+^ as the N–E–N interaction subtends smaller angles for longer E–N bonds.

**Fig. 3:**
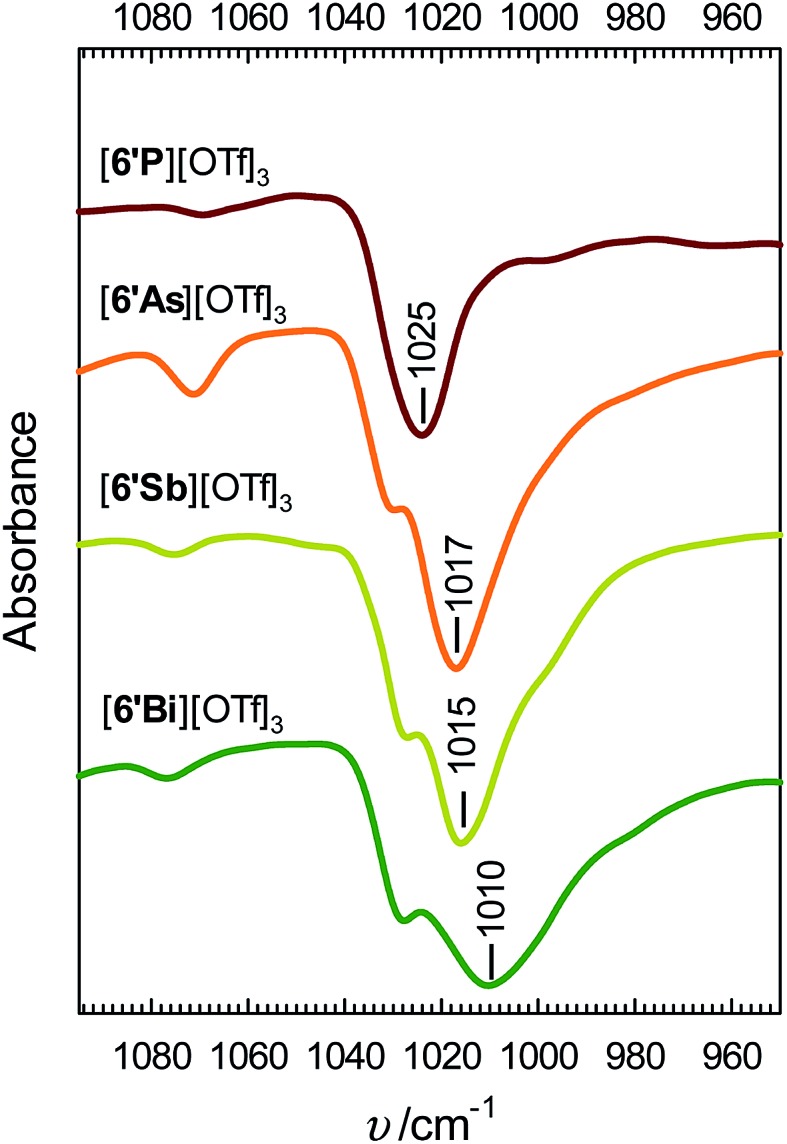
Infrared spectra (950–1100 cm^–1^) of [**6′E**][OTf]_3_ obtained on powdered salts using an ATR module.

Infrared spectra for derivatives of [**6′E**][OTf]_3_ enable quantification of the interion coordination in the solid state. The symmetric SO_3_ stretch, *ν*_s_(SO_3_), in several triflate salts has been studied previously and appears as a characteristically sharp absorbance in the 1020–1050 cm^–1^ range.^28^ The portions of the infrared spectra of [**6′E**][OTf]_3_ shown in Fig. 3 illustrate a trend in *ν*(SO_3_) of E = P > As > Sb > Bi, which we attribute to the degree of charge transfer from the anion to the pnictogen centre, which influences the S–O bond order. The broader bands for the heavier homologues are attributed to the loss of *C*_3v_ symmetry due to cation–anion interaction. The spectra for ligand-free Sb(OTf)_3_ and Bi(OTf)_3_, for which extensive Sb–O and Bi–O interactions are predicted, exhibit similarly broad S–O stretching bands (see Fig. S2, ESI†) that are shifted to lower frequencies (958 cm^–1^ and 1000 cm^–1^, respectively) than the analogous value in [Bu_4_N][OTf] (1032 cm^–1^),^28^ which features a weakly coordinating cation, and the calculated value for an isolated triflate anion in the gas phase (1035 cm^–1^, PBE0/aug-cc-pVTZ).

### Gas-phase structures, bonding, and Lewis acidity of [**6E**]^3+^

Optimized structures for [**6E**]^3+^ in the gas phase adopt a disphenoidal *C*_2_ symmetry for all derivatives, consistent with the observed solid-state structures. Selected calculated bond lengths and angles are given in Table 2. As observed experimentally in the solid state, the computed structures reveal axial E–N distances that are longer by *ca.* 0.1 Å than the equatorial E–N distances and average E–N bond distances that are primarily determined by the respective covalent radii. For pnictogen centers with a larger covalent radius, the bite angle N_1/3_–E–N_2/4_ and the equatorial and the axial interligand angles are smaller. The equatorial interligand angle in the solid-state structures of Sb and Bi derivatives are significantly smaller than in the anion-free gas-phase structures of [**6Sb**]^3+^ and [**6Bi**]^3+^, suggesting that the steric pressure of the interion contacts present in the solid state influence this angle. By comparison the axial interligand angle N_1_–E–N_3_ in the experimental and calculated structures are essentially identical, implying minimal distortion due to interion contacts.

**Table 2:** Select bond lengths and angles in the calculated (gas-phase, PBE0/def2-TZVP) structures of cations [**6E**]^3+^. See Fig. 2 for numbering scheme

Cation	N_1/3_–E	N_2/4_–E	N_1/3_–E–N_2/4_	N_2_–E–N_4_	N_1_–E–N_3_
[**6P**]^3+^	1.9511	1.8224	82.72	100.61	175.68
[**6As**]^3+^	2.0993	1.9714	78.86	97.39	168.47
[**6Sb**]^3+^	2.2545	2.1677	73.96	95.19	157.34
[**6Bi**]^3+^	2.3607	2.2678	71.50	95.12	153.59

Natural Bond Orbital (NBO) partial charges and Wiberg Bond Indices (WBI) for the gas-phase cations are listed in Table 3, evidencing a high positive charge for the central pnictogen centre, which is greater for heavier elements, as expected on the basis of relative electronegativities. Consistently, the WBIs for the N–E interaction has the trend P > As > Sb > Bi, implying a more ionic E–N bond for the heavier pnictogens. For a given derivative, the WBI value for the axial E–N interactions is smaller than the equatorial interactions, indicating less effective bonding along the N_1_–E–N_3_ axis than in the N_2_–E–N_4_ plane. Noting that the equatorial interligand angles range from 95 to 100° in all cases, we surmise that of the three mutually perpendicular p-orbitals that serve as acceptor orbitals at E^3+^, two are engaged by N_2_ and N_4_ in the equatorial plane, while the third accommodates two strained *trans* interactions involving N_1_ and N_3_.

**Table 3:** Calculated (PBE0/def2-TZVP) NBO partial charges and Wiberg bond indices for [**6E**]^3+^ in the gas phase. See Fig. 2 for numbering scheme

E	Charge (E)	Charge (N_1/3_)	Charge (N_2/4_)	WBI (N_1/3_–E)	WBI (N_2/4_–E)
P	+1.40	–0.53	–0.52	0.49	0.69
As	+1.58	–0.54	–0.54	0.43	0.61
Sb	+1.78	–0.55	–0.56	0.39	0.58
Bi	+1.86	–0.54	–0.55	0.36	0.50

**Scheme 2:**
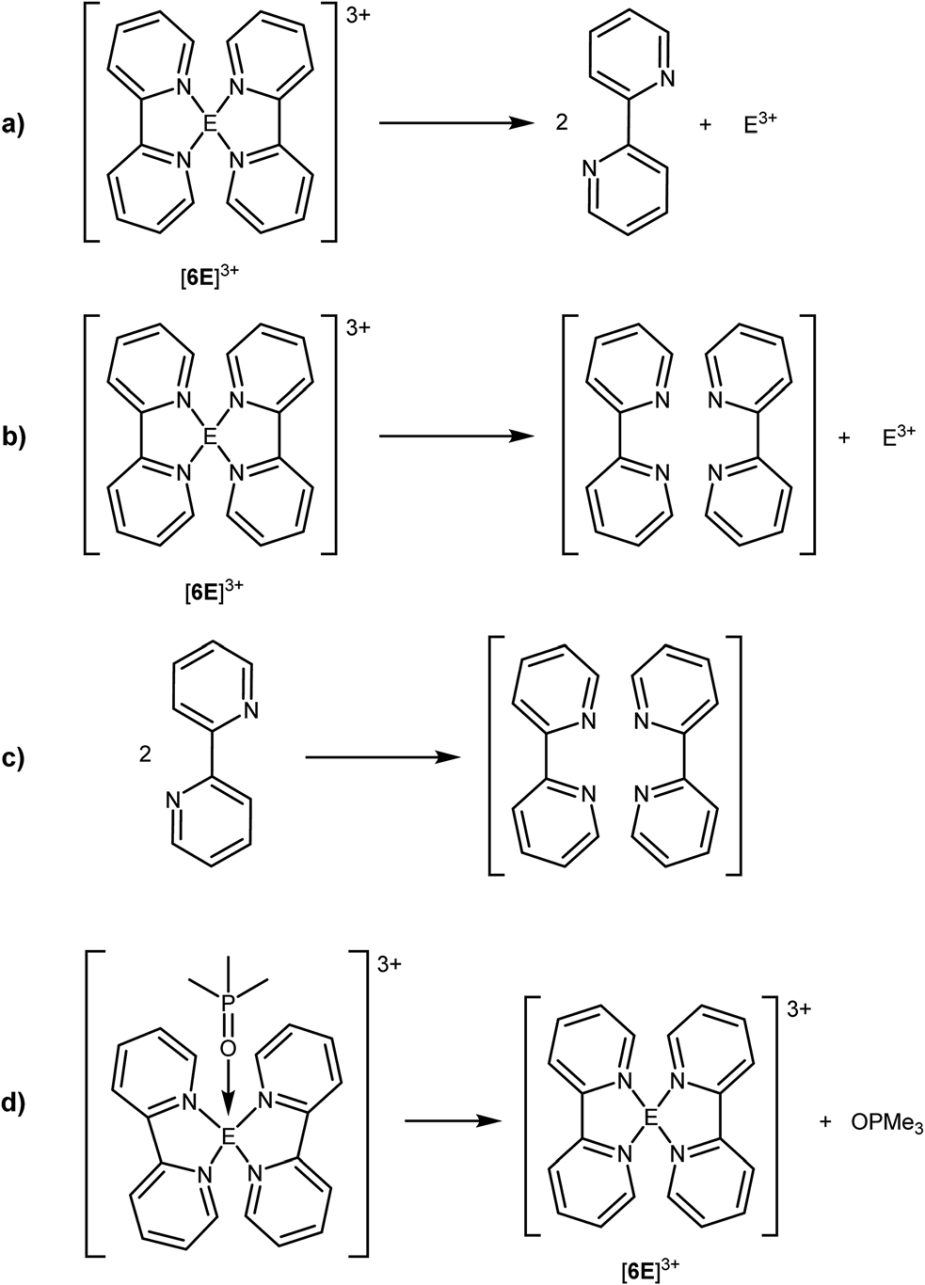
(a) Dissociation of two bipy ligands in [**6E**]^3+^, (b) dissociation of two bipy ligands in [**6E**]^3+^ with retention of the (bipy)_2_ geometry of [**6E**]^3+^, (c) organization of two bipy ligands to the (bipy)_2_ geometry found in [**6E**]^3+^, and (d) dissociation of OPMe_3_ from [(bipy)_2_E(OPMe_3_)]^3+^.

To assess the relative Lewis acidities of E^3+^, we have calculated the enthalpies for the heterolytic removal of both bipy ligands from [**6E**]^3+^. Scheme 2a represents removal of the ligands and relaxation of their geometries to the *C*_2h_ minimum for free bipy, and Scheme 2b represents removal of the ligands with retention of the geometry observed in [**6E**]^3+^. The difference between the two enthalpies represents the energy required for two non-interacting bipy molecules (*C*_2h_) to adopt the (bipy)_2_ geometry in each complex (Scheme 2c). The Δ*H*_rxn_ values in Table 4 show that the enthalpic requirement for ligand dissociation from E^3+^ has the trend E = Bi < Sb < As < P, irrespective of whether or not steric factors are considered. Values for ligand strain show a parallel trend, but the range (98–181 kJ mol^–1^) is small compared to the range for the overall ligand dissociation process (2302–3575 kJ mol^–1^). We therefore conclude that steric effects have a minor influence on the calculated enthalpies of ligand dissociation in Scheme 2a, which are dominated by electronic effects.

**Table 4:** Calculated (PBE0/def2-TZVP) reaction enthalpies (kJ mol^–1^, 298 K, gas phase) for the processes in Scheme 2a–d

E	Δ*H*_rxn_ (Scheme 2a)	Δ*H*_rxn_ (Scheme 2b)	Δ*H*_rxn_ (Scheme 2c)	Δ*H*_rxn_ (Scheme 2d)
P	3575	3756	181	178
As	3127	3264	137	210
Sb	2539	2649	110	244
Bi	2302	2400	98	255

We rationalize the calculated trend in dissociation enthalpies on the basis of atomic size, with a smaller atom having a higher charge concentration and the best orbital match in the N(sp^3^)→E(np) HOMO–LUMO interaction (*cf.* N(sp^3^)→P(3p) *vs.* As(4p) *vs.* Sb(5p) *vs.* Bi(6p)). The electrostatic and orbital interactions are both expected to weaken as atomic radii and the number of nodes in the acceptor p-orbitals increase. The trend in ligand strain is presumably related to the N_1_–E–N_3_ angle, which shows the most dramatic variation amongst all parameters in the calculated structures of [**6E**]^3+^, and decreases over a 22° range from phosphorus (175.68°) to bismuth (153.59°). We propose that the strained ligand geometry in [**6P**]^3+^ is enforced by orbital interactions involving three mutually perpendicular 3p acceptor orbitals at the P^3+^ centre. By comparison, in [**6Bi**]^3+^, where E–N bonding is calculated to be more ionic (Table 3), the preference for an N_1_–E–N_3_ angle of 180° is lowest.

While reaction enthalpies for Scheme 2a and b represent the Lewis acidity of monoatomic trications E^3+^, Δ*H*_rxn_ for Scheme 2d assesses the Lewis acidities of complexes [**6E**]^3+^ by measuring the energy required for removal of a prototypical ligand, OPMe_3_, from hypothetical complexes [(bipy)_2_E(OPMe_3_)]^3+^. The enthalpies for this process indicate that the Lewis acidity of complexes [**6E**]^3+^ has the trend E = P < As < Sb < Bi, which is the opposite trend to that of monoatomic E^3+^, and is rationalised on steric grounds acknowledging the trend in atomic radii and consequential coordination sphere. Consistently, the range of enthalpy values calculated for Scheme 2d (178–255 kJ mol^–1^) is much smaller than that observed for Scheme 2a (2302–3575 kJ mol^–1^) and is comparable to the ligand strain enthalpies calculated for Scheme 2c (98–181 kJ mol^–1^). In addition, comparison of the optimized structures for [**6E**]^3+^ and [(bipy)_2_E(OPMe_3_)]^3+^ shows that the greatest geometric deformation upon complexation with OPMe_3_ is compression of the interligand angle N_2_–E–N_4_ (see Fig. 2 for definition). The magnitude of this geometric adjustment, which leads to steric clash between the bipy ligands, is greatest for E = P (15°) and least for E = Bi (7°), consistent with the calculated trend for Scheme 2d.

### NMR characterization of [**6E**][OTf]_3_ and [**6′E**][OTf]_3_

CD_3_CN solutions of [**6E**][OTf]_3_ and [**6′E**][OTf]_3_ exhibit ^19^F NMR chemical shift values for all species in the range –78.9 to –79.5 ppm (*cf.* –79.4 for [PPh_4_][OTf]), indicative of dissociated triflate ions. In addition, solutions of all derivatives polymerize THF within hours of mixing, implicating a high Lewis acidity^29^,^30^ in coordinating solvents. No significant change was observed in the ^1^H or ^31^P NMR shifts of salts [**6′E**][OTf]_3_ over a broad concentration range, implying the absence of a bimolecular association process as might be expected from an equilibrium between the anion-bound and anion-free cations (see representative data for [**6′Bi**][OTf]_3_ in Fig. S3, ESI†). We conclude that CD_3_CN solutions of [**6E**][OTf]_3_ and [**6′E**][OTf]_3_ contain solvated trications and triflate anions with minimal interion interaction.

**Fig. 4:**
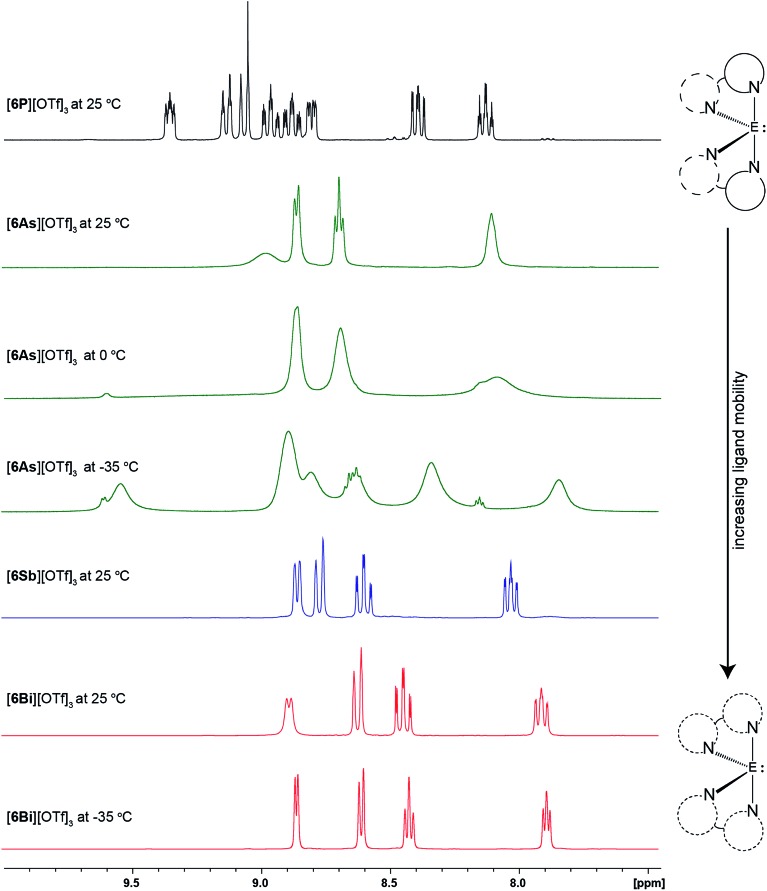
^1^H NMR resonances in the aromatic region for CD_3_CN solutions of [**6P**][OTf]_3_ (black), [**6As**][OTf]_3_ (green), [**6Sb**][OTf]_3_ (blue), and [**6Bi**][OTf]_3_ (red).

The aromatic resonances in the ^1^H NMR spectra of derivatives of [**6E**][OTf]_3_ in CD_3_CN are shown in Fig. 4. As predicted for a *C*_2_ symmetric bis-bipy complex, eight aromatic resonances are detected for [**6P**][OTf]_3_ at 25 °C. For [**6As**][OTf]_3_, four broad peaks are observed, which broaden further upon cooling to 0 °C and resolve into additional peaks upon cooling to –35 °C. Only four aromatic resonances are detected for [**6Sb**][OTf]_3_ and [**6Bi**][OTf]_3_, at 25 °C and at –35 °C. While the solid-state structure, featuring eight unique hydrogen environments for the cations in [**6E**][OTf]_3_ (Fig. 1), is apparently retained in solution for E = P, the mobility of the bipy ligands at 25 °C is sufficiently high for E = As, Sb, and Bi that only four hydrogen environments are detected. At –35 °C, ligand mobility is partially restricted for [**6As**][OTf]_3_ leading to additional signals but complete resolution of eight hydrogen environments, as in [**6P**][OTf]_3_, was not detected. The observations indicate a mobility for the bipy ligands around E with the trend E = P < As < Sb ≈ Bi and parallels the trend in ionicity for the E–N bond (Table 3). We propose that the more covalent N–P and N–As bonds are conformationally rigid due to the directional requirements of efficient orbital overlap to make a covalent bond (three mutually perpendicular p-orbitals), whereas the more ionic N–Sb and N–Bi interactions have a smaller barrier to motion due to the absence of a directional component for electrostatic interactions (point charges).

The difference between the ^31^P NMR chemical shift of free Et_3_PO and that of its adduct with a Lewis acid has been correlated with the strength of the Lewis acid (Gutmann–Beckett method).^31^,^32^ No systematic trend was observed in the chemical shifts observed (see Fig. S4, ESI†) in ^31^P NMR assays of solutions containing equimolar amounts of Et_3_PO and [**6′E**][OTf]_3_.^33^ Moreover, in the case of [**6′P**][OTf]_3_, a complex spectrum showing a mixture of products was obtained, none of which could be assigned to the phosphine oxide adduct. Deoxygenation of Et_3_PO by electrophilic phosphorus cations has been reported recently and may be operative.^34^ Moreover, as a wide range in covalent radii (1.11–1.51 Å)^25^ is spanned going from P to Bi, the steric influence on ^31^P chemical shifts may be greater than those due to differing Lewis acidities, confounding a straightforward assessment due to steric factors, as highlighted recently for borane Lewis acids.^35^

### Reactivity of [**6′P**][OTf]_3_ and [**6′As**][OTf]_3_

The structures of [**6P**][OTf]_3_ and [**6′P**][OTf]_3_ represent rare examples of hypervalent phosphorus(iii) acceptor centres, and are comparable to those involving N-heterocyclic carbene (NHC),^36^ phosphine,^37^,^38^ catecholate,^39^ and phenylpyrazole^40^ ligands. Moreover, electron precise (8 valence electron) phosphorus based frameworks **3** and **4** (Chart 1) are the only phosphorus(iii) centred trications that have been structurally characterized.^41^ By comparison, the 10 valence electron count imposed by the two chelate ligands at phosphorus in [**6P**]^3+^ and [**6′P**]^3+^ render these trications as novel examples of electron-rich phosphorus Lewis acids. Examples of arsenic(iii)-centred mono- and dications featuring phosphine^42^ or bipy^43^ ligands have been reported as well as two-coordinate arsenium monocations.^44^–^46^ However, [**6′As**][OTf]_3_ is the first structurally authenticated example of an arsenic-centred trication.

The reactivity of Sb(OTf)_3_ and Bi(OTf)_3_ has been studied previously, leading to their widespread use as Lewis acid catalysts,^19^,^20^ but the absence of synthetic routes to P(OTf)_3_ and As(OTf)_3_ has precluded investigations of these potential synthetic reagents. Phosphorus polycations have been used as a precursors to cationic bicyclophosphines and cyclic phosphorus oxides,^47^ and derivatives of **4** (Chart 1) have been shown to bind transition metal centers *via* the lone pair at the phosphorus(iii) center to give highly effective precatalysts for C–C bond forming reactions.^48^ Intrigued by the unique intersection of molecular and electronic structures represented by the trications in [**6′P**][OTf]_3_ and [**6′As**][OTf]_3_, and envisioning these salts as *in situ* equivalents of E(OTf)_3_ (E = P, As), we have conducted an initial survey of their reactivity.

### [**6′E**][OTf]_3_ as E(OTf)_3_ transfer reagents

**Scheme 3:**
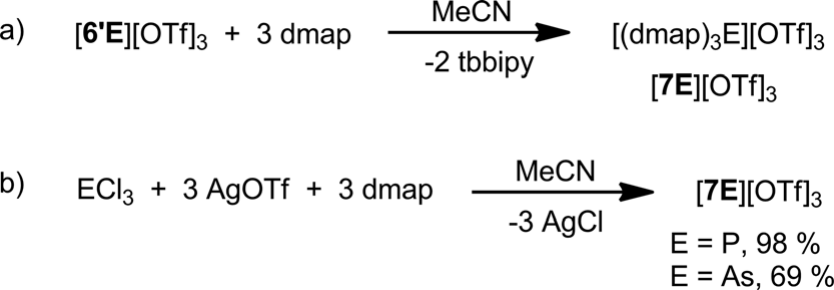
(a) Formation of [**7E**][OTf]_3_ from [**6′E**][OTf]_3_. (b) Independent synthesis of [**7E**][OTf]_3_ (E = P, As).

**Fig. 5:**
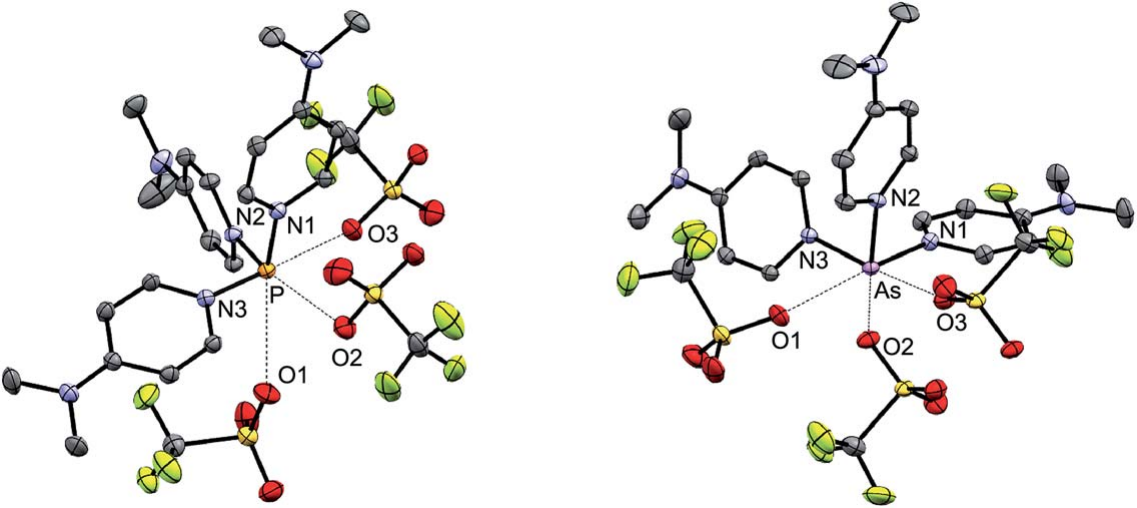
Solid state structures of [**7P**][OTf]_3_·1.5MeCN (left, one of two crystallographically distinct molecules shown) and [**7As**][OTf]_3_·2MeCN (right). Hydrogen atoms and solvent molecules have been omitted for clarity.

Reactions of [**6′E**][OTf]_3_ (E = P, As) with three equivalents of 4-dimethylaminopyridine (dmap) quantitatively (by ^31^P and ^1^H NMR) yield [(dmap)_3_E][OTf]_3_, [**7E**][OTf]_3_, (E = P, As) and free tbbipy (Scheme 3a). Neither dmap complexes could be isolated from the reaction mixtures but their identities were definitively established by independent syntheses (Scheme 3b) and structural elucidation (Fig. 5). While the [**7P**]^3+^ ion has previously been detected spectroscopically in mixtures of PCl_3_ and dmap,^49^,^50^ the ^31^P NMR chemical shift attributed to the trichloride salt was reported to vary widely (*δ* = 79–114 ppm) depending upon concentration, suggesting a dynamic process.^51^ By comparison, [**7P**][OTf]_3_ exhibits a ^31^P NMR chemical shift (*δ* = 101.7 ppm) for the redissolved crystals that does not vary over a broad concentration range.

The solid-state structures of [**7E**][OTf]_3_ reveal three dmap ligands bound to the pnictogen centers giving a trigonal pyramidal geometry at the pnictogen centre for the [**7E**]^3+^ ions.^52^ Three weak contacts with the triflate anions are evident, giving a six-coordinate geometry that is distorted by the presence of a stereochemically active lone pair in each case. The three weak E···O contacts are *trans* configured with respect to the three E–N bonds. The E–N bond lengths (Table 5) reflect the relative atomic radii of the phosphorus and arsenic atoms and are 0.1–0.2 Å shorter than the corresponding values in [**6P**][OTf]_3_, [**6′P**][OTf]_3_, and [**6′As**][OTf]_3_ due to the greater basicity and lesser steric demands of dmap compared to bipy or tbbipy. The N–E–N bond angles in [**7E**][OTf]_3_ are in the 90–100° range consistent with values observed for the *cis*-configured N–E–N angles in derivatives of [**6E**][OTf]_3_ and [**6′E**][OTf]_3_ (Table 5).

**Table 5:** Selected bond lengths (Å) and angles (°) in the solid-state structures of [**7P**][OTf]_3_·1.5MeCN and [**7As**][OTf]_3_·2MeCN

	[**7P**][OTf]_3_·1.5MeCN	[**7As**][OTf]_3_·2MeCN
E–N_1_	1.7635(17)	1.9157(17)
E–N_2_	1.7578(16)	1.9447(16)
E–N_3_	1.7588(17)	1.9174(16)
E–O_OTf_	3.0462(18)	2.8428(15)
	3.2615(17)	2.654(2)
	3.0395(19)	2.969(2)
N_1_–E–N_2_	98.42(8)	92.51(7)
N_2_–E–N_3_	97.40(8)	92.64(7)
N_1_–E–N_3_	99.24(8)	96.45(7)

### [**6′E**]^3+^ as synthons for E^I^ cations

**Scheme 4:**
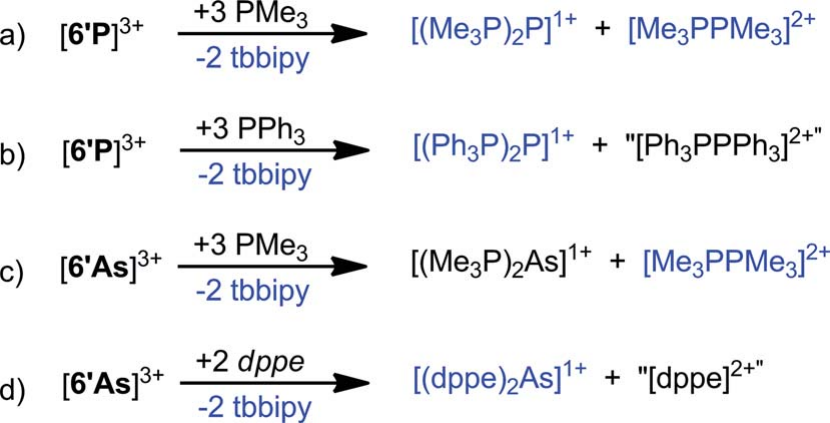
Reactions of [**6′E**][OTf]_3_ (E = P, As) with phosphines. Species in blue were definitively identified by their previously reported ^1^H or ^31^P NMR resonances.

In contrast to ligand exchange with dmap, reaction of [**6′P**][OTf]_3_ with PMe_3_ yields products due to redox chemistry (Scheme 4a). The previously reported P^I^ containing reduction product, [(Me_3_P)_2_P]^1+^ (*δ* = 15.0 and –156.3 ppm, ^1^*J*_PP_ = 438 Hz)^53^ and the P^IV^ containing oxidation product, [Me_3_PPMe_3_]^2+^ (*δ* = 28.4 ppm),^54^ have been definitively identified by ^31^P NMR spectroscopy (Fig. S5, ESI†). The analogous reaction with PPh_3_ yielded [(Ph_3_P)_2_P]^1+^ (*δ* = 30, –174, ^1^*J*_PP_ = 502 Hz)^55^ as the major product, but a complex mixture of oxidation products was obtained, suggesting that [Ph_3_PPPh_3_]^2+^, which is isoelectronic with the metastable hexaphenylethane molecule,^56^ may also be unstable relative to its constitutional isomers (Scheme 4b). Similarly, a ^31^P NMR assay of the 1 : 3 reaction between [**6′As**][OTf]_3_ and PMe_3_ showed a singlet due to [Me_3_PPMe_3_]^2+^ together with a resonance at 22.4 ppm, tentatively assigned to the As^I^ cation, [(Me_3_P)_2_As]^1+^, which could not be isolated from the reaction mixture (Scheme 4c). In a parallel experiment, a ^31^P NMR assay of the 1 : 2 reaction between [**6′As**][OTf]_3_ and 1,2-bis(diphenylphosphino)ethane (dppe) showed a singlet at 60.5 ppm due to the previously reported As^I^ cation [(dppe)As]^1+^,^57^ and unidentified oxidation products (Scheme 4d). We conclude that trications [**6′E**]^3+^ are strong oxidizing agents owing to their formidable molecular charge and effect oxidative P–P coupling while being reduced to P^I^ or As^I^ containing monocations. This redox outcome contrasts the ligand displacement observed in the presence of the more oxidatively resistant ligand dmap, and is analogous to reactivity patterns established for FSb(OTf)_2_ and Sb(OTf)_3_.^21^

### C–H and H–H bond activation by [**6′E**]^3+^

**Scheme 5:**
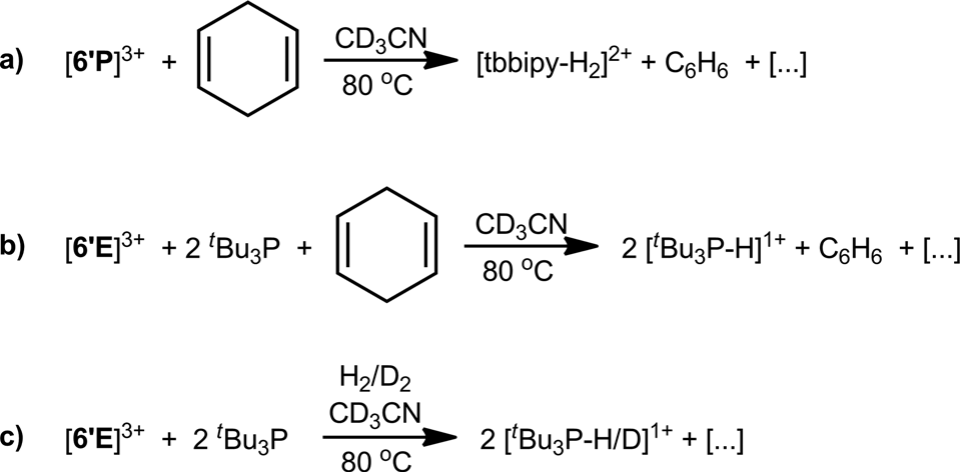
C–H and H–H bond activation by [**6′E**][OTf]_3_.

The equimolar reaction of [**6′P**][OTf]_3_ with 1,4-cyclohexadiene in CD_3_CN showed complete consumption of starting materials after 16 hours at 80 °C (Scheme 5a). The ^1^H NMR of the reaction mixture showed formation of benzene (*δ* = 7.38 ppm) and diprotonated tbbipy as the major products (>80%, Fig. S6, ESI†). The ^31^P NMR spectrum exhibits a mixture of unidentified products, none of which exhibit P–H couplings. The spectroscopic data are consistent with C–H bond activation involving dehydrogenation of 1,4-cyclohexadiene and sequestering of protons in [tbbipy-H_2_]^2+^. The analogous reaction with [**6′As**][OTf]_3_ showed only 10% conversion of 1,4-cyclohexadiene to benzene over 16 h at 80 °C, with concomitant formation of [tbbipy-H_2_]^2+^ and an insoluble black precipitate (Fig. S7, ESI†). C–H bond activation has recently been reported for mixtures of diphosphonium dications and ^*t*^Bu_3_P.^58^ Consistently, the 1 : 2 combinations of [**6′E**][OTf]_3_ (E = P, As) and ^*t*^Bu_3_P in CD_3_CN effect complete dehydrogenation of 1,4-cyclohexadiene to yield benzene and [^*t*^Bu_3_P-H]^1+^ within 16 hours at 80 °C (Scheme 5b). In line with the expectation that these reactions proceed *via* formation of a frustrated Lewis pair^59^ between [**6′E**]^3+^ and ^*t*^Bu_3_P, ^31^P NMR spectra of equimolar reaction mixtures containing ^*t*^Bu_3_P and either [**6′P**][OTf]_3_ or [**6′As**][OTf]_3_ show no evidence of coordination between the strong Lewis acids and the bulky base pairs (see Fig. S8, ESI†). Frustrated Lewis pair activity is also evidenced by 1 : 2 mixtures of [**6′P**][OTf]_3_ and ^*t*^Bu_3_P in CD_3_CN with H_2_ or D_2_ (1 atm pressure) in a sealed NMR tube at 80 °C over 16 hours (Scheme 5c), which show complete conversion of ^*t*^Bu_3_P to [^*t*^Bu_3_P-H]^1+^ or [^*t*^Bu_3_P-D]^1+^ by ^31^P NMR spectroscopy (Fig. S9, ESI†).

### [**6′E**]^3+^ as synthons for E^V^ cations

The ^31^P NMR spectrum of an equimolar mixture of [**6′P**][OTf]_3_ and SO_2_Cl_2_ shows a single ^31^P NMR resonance at *δ* = –146.9 ppm, assigned to the P^V^ containing [(tbbipy)_2_PCl_2_]^3+^. The upfield resonance is consistent with a five- or six-coordinate geometry and is similar to shifts reported for [(dmap)_2_PCl_4_]^1+^ (*δ* = –196 ppm)^60^ and [(bipy)PCl_4_]^1+^ (*δ* = –191 ppm).^61^ Moreover, the singlet at –146.9 ppm is also observed in the ^31^P NMR spectrum of a 2 : 1 : 3 mixture of tbbipy, PCl_5_ and TMSOTf.
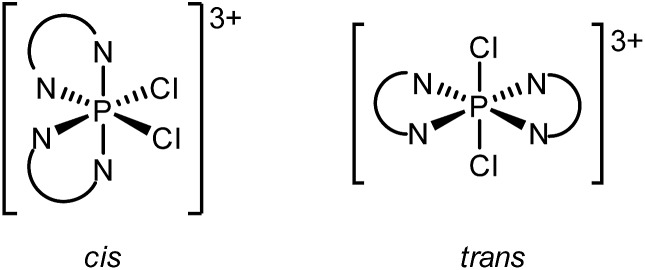


**Fig. 6:**
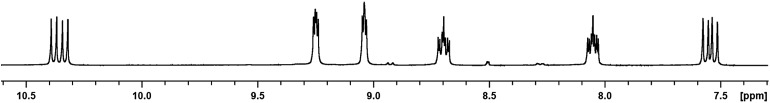
Portion of the ^1^H NMR spectrum (CD_3_CN, 298 K) of the crude reaction mixture containing equimolar amounts of [**6′P**][OTf]_3_ and SO_2_Cl_2_.

Two configurational outcomes are envisioned for the octahedral structure of [(tbbipy)_2_PCl_2_]^3+^, with a *cis* or *trans* arrangement of the chlorine atoms. The ^1^H NMR spectrum of the cation shows six resonances in the aromatic region (Fig. 6) and two resonances for the ^*t*^Bu groups, consistent with *C*_2_ symmetry, precluding a *trans* configuration of chlorine centres. Gas-phase calculations using bipy ligands revealed that both isomers are true energy minima (no negative vibrational frequencies), but a 64 kJ mol^–1^ preference for the *cis* isomer was calculated, arising from significant steric clash between the *ortho* hydrogen atoms of the ligands when a *trans* configuration is imposed (Fig. S10, ESI†). No *cis*/*trans* isomerism was detected experimentally upon heating a sample to 80 °C for an hour, consistent with the rigidity of the disphenoidal frame inferred for [**6P**]^3+^ from ^1^H NMR spectroscopy (Fig. 4).

Addition of excess Cl_2_ gas to MeCN solution of [**6′P**][OTf]_3_ yields a product with identical spectral features as those assigned to [(tbbipy)_2_PCl_2_]^3+^, as well as a number of unidentified byproducts. Interestingly, equimolar mixtures of [**6′As**][OTf]_3_ and SO_2_Cl_2_ showed no evidence of reaction even after heating to 80 °C for 2 hours. ^1^H NMR assays of these reaction mixtures showed only signals due to unreacted [**6′As**][OTf]_3_.

## Conclusions

In summary, we have isolated and comprehensively characterized the bipyridine complexes [**6E**][OTf]_3_ and [**6′E**][OTf]_3_ for E = P, As, Sb, Bi, representing rare examples of salts containing trications and unique homologous series. The solid-state structures show systematic variations as a function of the atomic size of E. Larger element centers facilitate interion interactions for [**6E**][OTf]_3_ and [**6′E**][OTf]_3_ in the order E = P < As < Sb < Bi as determined by X-ray crystallography and infrared spectroscopy. Gas-phase calculations (PBE0/def2-TZVP) reveal a trend from polar covalent to ionic E–N bonds for [**6E**]^3+^ going from E = P to E = Bi, consistent with data from ^1^H NMR spectroscopy. The Lewis acidity of monoatomic trications E^3+^ exhibits the trend E = Bi < Sb < As < P based on calculation of charge densities and ligand dissociation energies in the gas phase. However the calculated Lewis acidity of complexes [**6E**]^3+^ towards a prototypical ligand, OPMe_3_, exhibit the opposite trend, E = P < As < Sb < Bi due to steric factors.

**Scheme 6:**
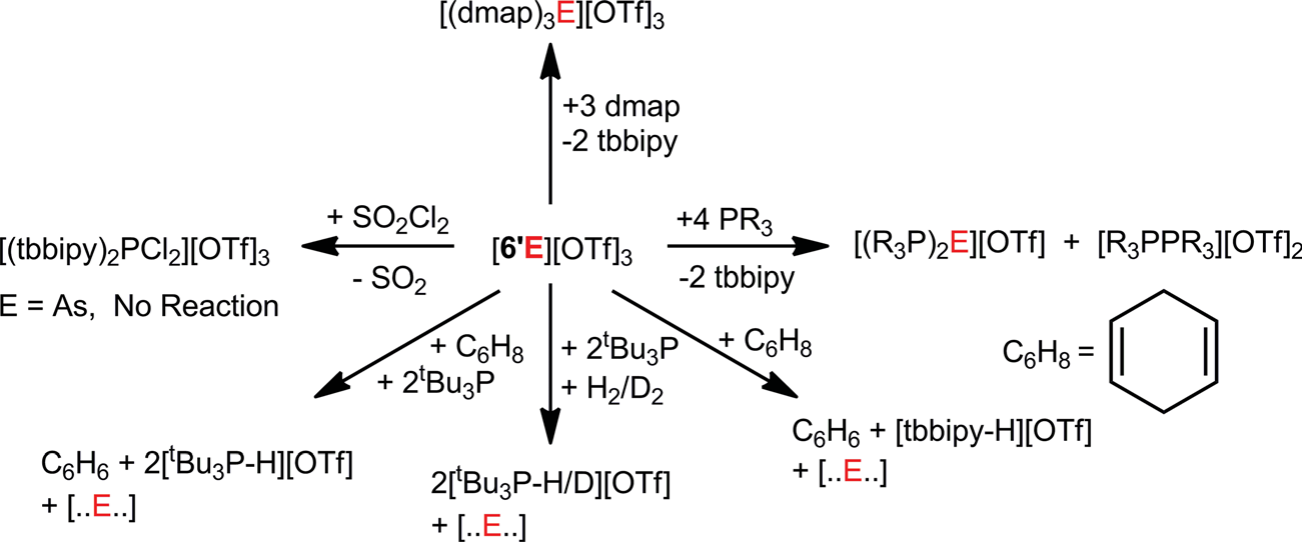
Reactivity of [**6′E**][OTf]_3_ (E = P, As).

Derivatives of [**6′E**][OTf]_3_ with E = P and As represent rare examples of non-metal triflates and E(OTf)_3_ transfer reagents, as illustrated by reactions with dmap, which proceed *via* ligand displacement to yield [(dmap)_3_E][OTf]_3_ and free tbbipy. Reactions of [**6′E**][OTf]_3_ with PR_3_ give access to E^I^-containing cations concomitant with oxidative P–P coupling. Cations [**6′E**]^3+^ (E = P, As) are single-component C–H bond activating agents as shown by dehydrogenation of 1,4-cyclohexadiene, which occurs more rapidly for E = P than for E = As. Both cations also dehydrogenate 1,4-cyclohexadiene in the presence of ^*t*^Bu_3_P, indicative of frustrated Lewis pair chemistry. Combinations of [**6′E**][OTf]_3_ (E = P, As) with ^*t*^Bu_3_P activate H_2_ or D_2_ under mild conditions to give [^*t*^Bu_3_P-H/D]^1+^. While the reaction of [**6′P**][OTf]_3_ with SO_2_Cl_2_ furnished the P^V^-containing [(tbbipy)_2_PCl_2_][OTf]_3_, the analogous oxidation of [**6′As**][OTf]_3_ was not observed. These observations highlight a rich reaction chemistry for P(OTf)_3_ and As(OTf)_3_ (Scheme 6) that is rendered accessible in salts [**6E**][OTf]_3_ and [**6′E**][OTf]_3_.

## Supplementary Material

Supplementary information

Crystal structure data
