# Correction: Bipyridine complexes of E^3+^ (E = P, As, Sb, Bi): strong Lewis acids, sources of E(OTf)_3_ and synthons for E^I^ and E^V^ cations

**DOI:** 10.1039/c6sc90005d

**Published:** 2016-01-11

**Authors:** Saurabh S. Chitnis, Alasdair P. M. Robertson, Neil Burford, Brian O. Patrick, Robert McDonald, Michael J. Ferguson

**Affiliations:** a Department of Chemistry , University of Victoria , Victoria , British Columbia V8W 3V6 , Canada . Email: nburford@uvic.ca ; Fax: +1 250 721 7147 ; Tel: +1 250 721 7150; b Department of Chemistry , University of British Columbia , Vancouver , British Columbia V6T 1Z1 , Canada; c Department of Chemistry , University of Alberta , Edmonton , Alberta T6G 2T2 , Canada

## Abstract

Correction for ‘Bipyridine complexes of E^3+^ (E = P, As, Sb, Bi): strong Lewis acids, sources of E(OTf)_3_ and synthons for E^I^ and E^V^ cations’ by Saurabh S. Chitnis *et al.*, *Chem. Sci.*, 2015, **6**, 6545–6555.



## 

Ref. 50 for the paper contained the incorrect year; the correct reference is as follows:

50 R. Weiss and S. Engel, *Angew. Chem., Int. Ed.*, 1992, **31**, 216–217.

The Royal Society of Chemistry apologises for these errors and any consequent inconvenience to authors and readers.

