# Supplementary material for: Bipyridine complexes of E3+ (E = P, As, Sb, Bi): strong Lewis acids, sources of E(OTf)3 and synthons for EI and EV cations
Source: Chem Sci. 2015 Aug 3;6(11):6545–55. doi: 10.1039/c5sc02423d (PMC6054056; doi:10.1039/c5sc02423d)
Supplement: Supplementary file 1 [file SC-006-C5SC02423D-s001.pdf]

This journal is © The Royal Society of Chemistry 2015

EXPERIMENTAL

Reagents and Apparatus. All reagents were handled in a MBraun Labmaster 130 glovebox (dry N_2) or on a grease-free dual manifold argon and vacuum (base vacuum = $2x10^{-2}$ mbar) Schlenk line. Solvents were dried by distillation from NaK (pentane, hexane, Et_2O) or CaH_2 (CH_2Cl_2). Anhydrous grade MeCN was obtained from Sigma-Aldrich and used without distillation. Prior to use all solvents were stored for at least 48 h over 3 Å molecular sieves, which had been freshly activated at 300 ºC under dynamic vacuum for 24 h. PCl_3 (99 %), $AsCl_3$ (99.99 %), $BiCl_3$ (99.99 %), *bipy* (> 99 %), and *tbbipy* (98 %) were obtained from Sigma-Aldrich and used without further purification. SbF_3 (98 %) was obtained from Sigma-Aldrich and sublimed prior to use. Deuterated solvents were dried using the same procedure as MeCN. PMe_3 (97%) and TMSOTf (99%) were distilled before use. Reactions were carried out inside the glovebox in screw-cap glass vials that had been dried at 200 ºC for at least 1 h and placed under dynamic vacuum (glovebox antechamber) while still hot. NMR tubes fitted with J-Young valves were charged and sealed inside the glovebox. Infrared spectra were obtained on a Perkin Elmer Frontier instrument equipped with a diamond ATR module. Elemental analyses were carried out by Canadian Microanalytical Ltd. in Delta, British Columbia, Canada. All quantum chemical calculations were carried out using Gaussian 09.[1]

General procedure for [6E][OTf]$_3$: ECl_3 (1 mmol) and AgOTf (3 mmol, 0.772 g) were combined in 12 mL MeCN and stirred in the dark for 1 hour. Solid *bipy* (2 mmol, 0.312 g) was added in three portions over 5 minutes and the resulting suspension was allowed to stir for an additional hour. The suspension was filtered to remove AgCl, concentrated to 3 mL and recrystallized at -30 °C. Products were isolated by decanting the mother liquor and removing all volatiles under vacuum. Characterization data is given below.

[1] Gaussian 09, Revision **D.01**, M. J. Frisch, G. W. Trucks, H. B. Schlegel, G. E. Scuseria, M. A. Robb, J. R. Cheeseman, G. Scalmani, V. Barone, B. Mennucci, G. A. Petersson, H. Nakatsuji, M. Caricato, X. Li, H. P. Hratchian, A. F. Izmaylov, J. Bloino, G. Zheng, J. L. Sonnenberg, M. Hada, M. Ehara, K. Toyota, R. Fukuda, J. Hasegawa, M. Ishida, T. Nakajima, Y. Honda, O. Kitao, H. Nakai, T. Vreven, J. A. Montgomery, Jr., J. E. Peralta, F. Ogliaro, M. Bearpark, J. J. Heyd, E. Brothers, K. N. Kudin, V. N. Staroverov, R. Kobayashi, J. Normand, K. Raghavachari, A. Rendell, J. C. Burant, S. S. Iyengar, J. Tomasi, M. Cossi, N. Rega, J. M. Millam, M. Klene, J. E. Knox, J. B. Cross, V. Bakken, C. Adamo, J. Jaramillo, R. Gomperts, R. E. Stratmann, O. Yazyev, A. J. Austin, R. Cammi, C. Pomelli, J. W. Ochterski, R. L. Martin, K. Morokuma, V. G. Zakrzewski, G. A. Voth, P. Salvador, J. J. Dannenberg, S. Dapprich, A. D. Daniels, Ö. Farkas, J. B. Foresman, J. V. Ortiz, J. Cioslowski, and D. J. Fox, Gaussian, Inc., Wallingford CT, 2009.

[(*bipy*)$_2$P][OTf]$_3$: Yield: 0.317 g (40 %); Melting Point: 237 °C dec.; Elemental Analysis (calcd./expt.): C (34.94/34.86), H(2.04/2.18), N(7.09/7.72); IR (ATR, 298 K, [cm^{-1}]): 316 (m), 420 (s), 428 (m), 441 (m), 451 (s), 490 (w), 506 (s), 515 (vs), 573 (s), 633 (vs), 687 (m), 713 (m), 720 (m), 758 (w), 767 (m), 785 (s), 908 (vw), 1012 (vs), 1024 (vs), 1046 (vw), 1072 (w), 1109 (w), 1142 (s), 1159 (s), 1223 (vs), 1247 (vs), 1322 (w), 1377 (vw), 1432 (w), 1451 (m), 1471 (m), 1506 (w), 1537 (vw), 1571 (vw), 1616 (m), 2255 (w), 3051 (w), 3103 (w); ^{1}H NMR (CD_3CN, 298 K, 300.15 MHz): 1.97 (*singlet*, 3H), 8.13 (*pseudo multiplet of triplets*, 2H), 8.39 (*pseudo multiplet of triplets*, 2H), 8.78-8.81 (*multiplet*, 2H), 8.88 (*pseudo multiplet of triplets*, 2H), 8.96 (*pseudo multiplet of triplets*, 2H), 9.06 (*doublet*, $^3J_{HH}$ = 8.2 Hz), 9.13 (*doublet of triplets*, $^3J_{HH}$ = 8.1 Hz, 1.4 Hz); ^{13}C{^{1}H} (CD_3CN, 298 K, 75.5 MHz, [ppm]): 126.9 (*doublet*, 6 Hz), 127.8 (*doublet*, 4 Hz), 131.8 (*doublet*, 4 Hz), 133.0 (*doublet*, 6 Hz), 145.5 (*doublet*, 13 Hz), 148.8 (*singlet*), 148.9 (*doublet*, 11 Hz), 152.5 (*singlet*) ; ^{31}P NMR (CD_3CN, 298 K, 202.5 MHz, δ [ppm]): 33.94 (*singlet*), ^{19}F{^{1}H} NMR (CD_3CN, 298 K, 282.5 Hz, δ [ppm]): -79.31 (*singlet*).

[(*bipy*)$_2$As][OTf]$_3$: Yield: 0.813 g (97 %); Melting Point: 254-262 °C dec.; ; Elemental Analysis (calcd./expt.): C (33.10/33.15), H(1.93/2.02), N(6.71/6.65); IR (ATR, 298 K, [cm^{-1}]): 225 (m), 244 (m), 262 (m), 277 (w), 291 (m), 302 (s), 319 (w), 352 (m), 409 (m), 418 (m), 461 (m), 516 (s), 574 (m), 633 (vs), 666 (w), 723 (m), 752 (vw), 756 (vw), 764 (m), 777 (s), 805 (w), 849 (vw), 904 (vw), 1008 (s), 1024 (s), 1063 (w), 1075 (w), 1153 (s), 1162 (s), 1203 (m), 1225 (m), 1261 (m), 1276 (m), 1297 (w), 1322 (vw), 1334 (vw), 1429 (w), 1451 (m), 1474 (w), 1505 (w), 1572 (vw), 1606 (m), 1631 (vw), 3069 (vw), 3096 (w), 3125 (vw); ^{1}H NMR (CD_3CN, 298 K, 300.15 MHz): 7.96 (*broad singlet*, 1H), 8.35 (*broad singlet*, 1H), 8.49 (*broad singlet*, 1H), 8.77 (*broad singlet*, 2H), 8.91 (*broad singlet*, 1H), 8.93 (*broad singlet*, 1H), 9.49 (*broad singlet*, 1H); ^{13}C{^{1}H} (CD_3CN, 298 K, 75.5 MHz, [ppm]): 126.0 (*singlet*), 131.2 (*singlet*), 147.4 (*singlet*), 148.0 (*singlet*), 148.2 (*singlet*); ^{19}F{^{1}H} NMR (CD_3CN, 298 K, 282.5 Hz, δ [ppm]): -79.4 (*singlet*).

[(*bipy*)$_2$Bi][OTf]$_3$•MeCN: Yield = 0.756 g (75 %); Melting Point: 220 °C dec.; Elemental Analysis with one molecule of MeCN (calcd./expt.): C (29.74/29.54) H(1.90/1.68) N(6.94/6.75); IR (ATR, 298 K, [cm^{-1}]): 227 (m), 261 (w), 317 (m), 353 (s), 410 (m), 438 (vw), 470 (w), 514 (vs), 574 (s), 625 (vs), 633 (vs), 653 (w), 727 (m), 765 (s), 807 (w), 837 (vw), 1005 (vs), 1031 (m), 1063 (w), 1074 (w), 1106 (m), 1157 (vs), 1207 (s), 1225 (s), 1256 (s), 1275 (s), 1322 (m), 1379 (vw), 1442 (m), 1475 (w),

1498 (w), 1567 (w), 1576 (w), 1598 (m), 1647 (m), 3040 (vw), 3086 (w), 3118 (w), 3275 (w); ^{1}H NMR (CD_3CN, 298 K, 300.15 MHz): 1.97 (*singlet*, 1H), 7.94 (*triplet*, 6.7 Hz, 2H). 8.47 (*triplet of doublets*, 7.5 Hz, 1.5 Hz, 2H), 8.64 (*doublet*, 8.2 Hz, 2H), 8.90 (*singlet*, 2H); $^{13}C\{^1H\}$ (CD_3CN, 298 K, 75.5 MHz, [ppm]): 121.0 (*quartet*, $^1J_{CF}$ = 320 Hz), 127.6 (*singlet*), 130.6 (*singlet*), 144.9 (*singlet*), 151.6 (*singlet*), 154.2 (*singlet*); $^{19}F\{^1H\}$ NMR (CD_3CN, 298 K, 282.5 Hz, δ [ppm]): -79.1 (*singlet*).

Synthesis of [(*tbbipy*)$_2$P][OTf]$_3$•MeCN: To a rapidly stirred suspension of PCl_3 (5.00 mmol, 0.687 g) and *tbbipy* (10.00 mmol, 2.684 g) in five portions to yield a yellow suspension. The suspension was allowed to stir for 2 hours and allowed to settle. The orange supernatant was separated via filtration and concentrated *in vacuo* to ca. 10 mL when a large amount of yellow precipitate appeared. The precipitate was allowed to settle over 48 hours at -30 °C and separated by decanting the light orange supernatant. The precipitate was washed with Et_2O (10 mL), filtered, and dried under dynamic vacuum for 8 hours to yield the product as a fine yellow powder. Yield: 3.560 g (70 %); Melting Point: 257 °C dec.; Elemental Analysis with one molecule of MeCN (calcd./expt.): C (46.63/46.70), H (4.87/5.28), N (6.63/6.34); IR (ATR, 298 K, [cm^{-1}]): 238 (w), 279 (vw), 298 (w), 315 (vw), 340 (vw), 418 (vw), 455 (m), 493 (m), 517 (s), 539 (w), 550 (w), 574 (s), 612 (w), 634 (vs), 697 (vw), 727 (w), 740 (w), 758 (w), 846 (s), 901 (m), 932 (m), 969 (w), 1025 (vs), 1071 (w), 1118 (w), 1163 (s), 1219 (s), 1232 (s), 1257 (s), 1293 (m), 1372 (w), 1385 (w), 1464 (w), 1483 (w), 1515 (w), 1543 (vw), 1592 (m), 1615 (m); ^{1}H NMR (CD_3CN, 298 K, 500 MHz, δ [ppm]): 1.50 (*singlet*, 18H), 1.58 (*singlet*, 18H), 8.04 (*pseudo doublet of triplets*, 6.9 Hz, 1.7 Hz, 2H), 8.34 (*doublet of doublets*, 6.3 Hz, 1.7 Hz, 2H), 8.64 (*doublet of doublets*, 6.9 Hz, 2.3 Hz, 2H), 9.02 (*multiplet* ,4H), 9.17 (*doublet of doublets*, 6.3 Hz, 2.9 Hz, 2H); $^{13}C\{^1H\}$ NMR (CD_3CN, 298 K, 125.79 MHz, δ [ppm]): 30.4 (*singlet*), 30.8 (*singlet*), 38.8 (*singlet*), 39.2 (*singlet*), 122.3 (*quartet*, $^1J_{CF}$ = 321 Hz), 125.0 (*doublet*, J_{CP} = 113 Hz), 124.9 (*doublet*, J_{CP} = 113 Hz), 129.6 (*doublet,* J_{CP} = 177.4 Hz), 129.5 (*doublet*, J_{CP} = 177.4 Hz), 143.8 (*doublet*, J_{CP} = 3 Hz), 144.6 (*doublet*, J_{CP} = 13 Hz), 147.4 (*singlet*), 148.1 (*singlet*), 175.1 (*doublet*, J_{CP} = 4 Hz), 179.3 (*doublet*, J_{CP} = 3 Hz); ^{31}P NMR (CD_3CN, 298 K, 202.5 MHz, δ [ppm]): 30.7 (*singlet*); $^{19}F\{^1H\}$ NMR (CD_3CN, 298 K, 282.5 Hz, δ [ppm]): -79.2 (*singlet*).

Synthesis of [(*tbbipy*)$_2$As][OTf]$_3$•MeCN: $AsCl_3$ (1 mmol, 0.181 g) and AgOTf (3 mmol, 0.771 g) were combined in 5 mL MeCN and stirred for 5 minutes. Solid *tbbipy* (2 mmol, 0.537 g) was added in five small portions and the resulting white suspension was allowed to stir for 4 hours and then filtered. The clear and colourless filtrate was concentrated to 3 mL, layered with Et_2O and placed in the freezer at -

30 °C for 16 hours to obtain white crystalline material. The supernatant was decanted and the solids were washed twice with 2 mL Et_2O. Removal of all volatiles under vacuum gave the product as a fine white powder. Yield: 0.677 g, 64 %; Melting Point: 245 °C dec.; Elemental Analysis (calcd./expt.): C (44.24/43.91), H (4.57/4.86), N (5.29/5.26); IR (ATR, 298 K, [cm^{-1}]): 210 (s), 219 (s), 236 (w), 251 (w), 292 (w), 330 (w), 350 (w), 368 (w), 414 (w), 483 (w), 516 (s), 545 (m), 573 (m), 602 (m), 634 (vs), 720 (w), 742 (w), 755 (w), 846 (m), 896 (m), 936 (w), 1017 (vs), 1071 (w), 1154 (s), 1210 (s), 1229 (s), 1252 (s), 1277 (s), 1370 (w), 1421 (m), 1484 (w), 1545 (w), 1614 (m); ^{1}H NMR (CD_3CN, 298 K, 500 MHz, [ppm]): 1.52 (*broad singlet*, 18H), 7.68-8.71 (*broad overlapping multiplet*, 3H), 2.32 (*singlet*, 2H), 8.98-9.76 (*broad singlet*, 1H); $^{13}C\{^{1}H\}$ (CD_3CN, 298 K, 125.81 MHz, [ppm]): 29.5 (*singlet*), 37.4 (*singlet*), 120.8 (*quartet*, $^{1}J_{CF}$ = 322 Hz), 123.9 (*singlet*), 127.9 (*broad singlet*), 146.6 (*singlet*), 147.2 (*broad singlet*), 173.6 (*broad singlet*); $^{19}F\{^{1}H\}$NMR (CD_3CN, 298 K, 282.5 Hz, [ppm]): -79.3 (*singlet*). Crystals suitable for diffraction were obtained from a MeCN solution and exhibited the same NMR resonances as the bulk material.

Synthesis of [(*tbbipy*)$_2$Sb][OTf]$_3$•MeCN: $Sb(OTf)_3$ (1 mmol, 0.569 g) and *tbbipy* (2 mmol, 0.537 g) were combined in 5 mL CH_2Cl_2 to obtain a clear and colourless solution which was stirred for 2 hours. Upon filtration, the colourless filtrate was concentrated to 2 mL under vacuum, layered with pentane, and placed in the freezer at -30 °C for 72 hours to obtain a fine white powder. Yield: 0.857 g, 77 %; Melting Point: 159-163 °C dec.; Elemental Analysis (calcd./expt.): C (42.36/41.41), H (4.38/4.09), N(5.07/5.03); IR (ATR, 298 K, [cm^{-1}]): 220 (vw), 228 (vw), 236 (vw), 259 (vw), 316 (w), 350 (w), 396 (w), 515 (s), 548 (w), 573 (m), 605 (m), 634 (vs), 719 (w), 849 (m), 898 (m), 1015 (vs), 1153 (s), 1199 (s), 1223 (s), 1262 (s), 1369 (w), 1416 (m), 1483 (w), 1546 (w), 1614 (m); ^{1}H NMR (CD_2Cl_2, 298 K, 300.15 MHz): 1.47 (*broad singlet*, 18H), 8.03 (*doublet of doublets*, J_{HH} = 6.3 Hz, 1.9 Hz, 4H), 8.68 (*broad doublet*, J_{HH} = 1.9 Hz, 4H), 8.71 (*broad doublet*, J_{HH} = 6.3 Hz, 4H); $^{13}C\{^{1}H\}$ (CD_2Cl_2, 298 K, 75.5 MHz, [ppm]): 29.8 (*singlet*), 36.6 (*singlet*), 119.9 (*quartet*, $^{1}J_{CF}$ = 323 Hz), 122.3 (*broad singlet*), 126.8 (*broad singlet*), 147.0 (*broad singlet*), 148.4 (*singlet*), 170.8 (*broad singlet*); $^{19}F\{^{1}H\}$ NMR (CD_2Cl_2, 298 K, 282.5 Hz, [ppm]): -78.9 (*singlet*).

Synthesis of [(*tbbipy*)$_2$Bi][OTf]$_3$: $BiCl_3$ (1 mmol, 0.316 g) and AgOTf (3 mmol, 0.771 g) were combined in a vial with 5 mL MeCN and stirred in the dark for 5 minutes to obtain a white suspension. Solid *tbbipy* (2 mmol, 0.537 g) was added in 5 portions and the suspension was stirred in the dark for 1 hour and then allowed to settle. The suspension was filtered and the precipitate was washed with an

additional 2 mL MeCN. The filtrates were combined and all volatiles were removed under vacuum to obtain the product as a fine white powder. Yield: 1.240 g, quantitative; Melting Point:131-132 °C; Elemental analysis was not obtained; IR (ATR, 298 K, [cm^{-1}]): 248 (m), 264 (vw), 297 (w), 318 (w), 356 (m), 393 (w), 420 (vw), 484 (vw), 515 (s), 545 (m), 573 (m), 604 (m), 631 (vs), 667 (vw), 692 (vw), 716 (w), 740 (w), 754 (w), 847 (m), 896 (m), 1010 (vs), 1150 (s), 1198 (s), 1222 (s), 1252 (s), 1262 (s), 1310 (m), 1368 (w), 1411 (m), 1444 (vw), 1467 (w), 1485 (w), 1548 (m), 1610 (m), 2873 (w), 2907 (w), 2967 (w); ^{1}H NMR (CD_2Cl_2, 298 K, 300.15 MHz): 1.44 (*singlet*, 36 H), 7.95 (*broad singlet*, 2H), 7.96 (*broad singlet*, 2H), 8.55 (*broad singlet*, 2H), 8.56 (*broad singlet*, 2H), 8.75 (*broad singlet*, 2H), 8.77 (*broad singlet*, 2H); $^{13}C\{^{1}H\}$ (CD_3CN, 298 K, 75.5 MHz, [ppm]): 30.3 (*singlet*), 37.3 (*singlet*), 121.8 (*quartet*, $^{1}J_{CF}$ = 319 Hz), 124.3 (*singlet*), 127.1 (*singlet*), 150.6 (*singlet*), 153.8 (*singlet*), 169.7 (*singlet*); $^{19}F\{^{1}H\}$ NMR (CD_2Cl_2, 298 K, 282.5 Hz, [ppm]): -79.1 (*singlet*).

Synthesis of [(*dmap*)$_3$P][OTf]$_3$: PCl_3 (1 mmol, 0.137 g) and AgOTf (3 mmol, 0.771 g) were combined in a vial with 4 mL MeCN and stirred in the dark to obtain a clear solution. Solid *dmap* (3 mmol, 0.367 g) was added in three portions with stirring to obtain a white suspension, which was stirred for 1 hour in the dark and allowed to settle. A clear and colourless solution was obtained upon filtration. Removal of all volatiles gave the product as an off white powder. Yield: 0.798 g, 98 %; Melting Point: 155 °C, dec.; Elemental analysis was precluded by samples decomposition under high vacuum, see ^{1}H, ^{31}P and ^{13}C NMR spectra below; IR (ATR, 298 K, [cm^{-1}]): 227 (m), 247 (w), 285 (vw), 315 (w), 347 (vw), 377 (m), 469 (m), 513 (s), 572 (s), 602 (s), 631 (vs), 692 (vw), 754 (w), 800 (m), 827 (m), 894 (vw), 942 (w), 998 (vs), 1000 (vs), 1070 (w), 1148 (s), 1214 (s), 1233 (m), 1263 (m), 1317 (w), 1346 (vw), 1405 (w), 1442 (w), 1497 (vw), 1566 (m), 1588 (w), 1636 (m); ^{1}H NMR (CD_3CN, 298 K, 300.15 MHz): 3.31 (*singlet*, 18H), 7.06 (*pseudo doublet*, 6H), 7.72 (*pseudo doublet*, 6 H); $^{13}C\{^{1}H\}$ (CD_3CN, 298 K, 75.5 MHz, [ppm]): 41.6 (*singlet*), 118.6 (*singlet*), 122.0 (*quartet*, $^{1}J_{CF}$ = 322 Hz), 142.9 (*singlet*), 158.8 (*singlet*); ^{31}P NMR (CD_3CN, 298 K, 121.6 MHz, δ [ppm]): 101.74 (*singlet*); $^{19}F\{^{1}H\}$ NMR (CD_2Cl_2, 298 K, 282.5 Hz, [ppm]): -79.1 (*singlet*). Colourless crystalline blocks were obtained upon storing a saturated MeCN solution for 48 hours and exhibited the same NMR spectra as the bulk material.

Synthesis of [(*dmap*)$_3$As][OTf]$_3$: $AsCl_3$ (1 mmol, 0.181 g) and AgOTf (3 mmol, 0.772 g) were combined in a vial and MeCN (4 mL) was added to obtain a clear and colourless solution. Solid *dmap* (3 mmol, 0.371 g) was added in three portions to obtain a white suspension, which was stirred in the dark for 30 minutes. The suspension was filtered and placed in the freezer at -30 °C overnight to obtain

colourless crystalline blocks, which were isolated by decanting the supernatant and dried under vacuum. Yield: 0.621 g, 69%; Melting Point: 162 °C dec.; Satisfactory elemental analysis could not be obtained, see ^{1}H and ^{13}C NMR spectra below; IR (ATR, 298 K, [cm^{-1}]): 228 (s), 247 (m), 253 (m), 284 (w), 313 (m), 349 (m), 377 (s), 469 (s), 511 (vs), 571 (s), 601 (s), 630 (vs), 692 (vw), 717 (vw), 744 (vw), 754 (w), 801 (m), 829 (m), 899 (vw), 998 (vs), 1010 (vs), 1069 (w), 1146 (vs), 1213 (s), 1232 (m), 1263 (m), 1319 (w), 1346 (vw), 1407 (w), 1443 (w), 1497 (w), 1566 (m), 1587 (w), 1638 (m), 2971 (vw), 3089 (vw), 3272 (w); ^{1}H NMR (CD_3CN, 298 K, 300.15 MHz): 3.23 (*singlet*, 18H), 6.93 (*pseudo doublet*, 6H), 8.06 (*pseudo doublet*, 6H); ^{13}C{^{1}H} (CD_3CN, 298 K, 75.5 MHz, [ppm]): 41.1 (*singlet*), 118.4 (*singlet*), 143.6 (*singlet*), 158.3 (*singlet*); ^{19}F{^{1}H} NMR (CD_2Cl_2, 298 K, 282.5 Hz, [ppm]): -79.9 (*singlet*).

Synthesis of [(*tbbipy*)$_2$PCl$_2$][OTf]$_3$: To a yellow suspension of [(*tbbipy*)$_{6'P}$][OTf]$_3$ (1 mmol, 1.016 g) in 3 mL MeCN was dropwise added a solution of SO_2Cl_2 (1 mL of a 1 M CH_2Cl_2 solution) over 1 minute. A clear yellow solution was obtained after stirring for 1 hour. Removal of all volatiles under dynamic vacuum gave a fine light yellow powder. Yield: 1.069 g, 99 %; Melting Point: °C. 204 °C dec.; Elemental Analysis (calcd./expt.): C (43.14/42.41), H(4.46/4.21), N (5.16/5.19); IR (ATR, 298 K, [cm^{-1}]): 226 (m), 302 (vw), 348 (w), 375 (vw), 422 (vw), 476 (m), 494 (s), 515 (vs), 528 (m), 549 (m), 572 (s), 604 (s), 635 (vs), 731 (w), 754 (w), 802 (vw), 842 (vw), 849 (m), 901 (m), 935 (w), 1026 (vs), 1069 (m), 1149 (s), 1222 (s), 1248 (s), 1373 (w), 1433 (m), 1487 (vw), 1540 (w), 1552 (w), 1618 (m), 2973 (w); ^{1}H NMR (CD_3CN, 298 K, 300.15 MHz): 1.42 (*singlet*, 18H), 1.69 (*singlet*, 18H), 7.54 (*pseudo doublet of doublets*, 2H), 8.05 (*multiplet*, 2H), 8.69 (*multiplet*, 2H), 9.03 (*pseudo triplet*, 2H), 9.24 (*pseudo doublet of doublets*, 2H), 10.35 (*pseudo doublet of doublets*, 2H); ^{13}C{^{1}H} (CD_3CN, 298 K, 75.5 MHz, [ppm]): 30.2 (*singlet*), 30.5 (*singlet*), 39.1 (*singlet*), 39.9 (*singlet*), 126.6 (*doublet*, 5 Hz), 127.1 (*doublet*, 5 Hz), 131.3 (*doublet*, 6 Hz), 131.6 (*doublet*, 7 Hz), 137.5 (*doublet*, 11 Hz), 139.1 (*doublet*, 11 Hz), 141.2 (*doublet*, 4 Hz), 145.3 (*doublet*, 5 Hz), 178.1 (*singlet*), 181.0 (*singlet*); ^{31}P NMR (CD_3CN, 298 K, 121.6 MHz, δ [ppm]): -146.88 (*singlet*); ^{19}F{^{1}H} NMR (CD_2Cl_2, 298 K, 282.5 Hz, [ppm]): -79.1 (*singlet*).

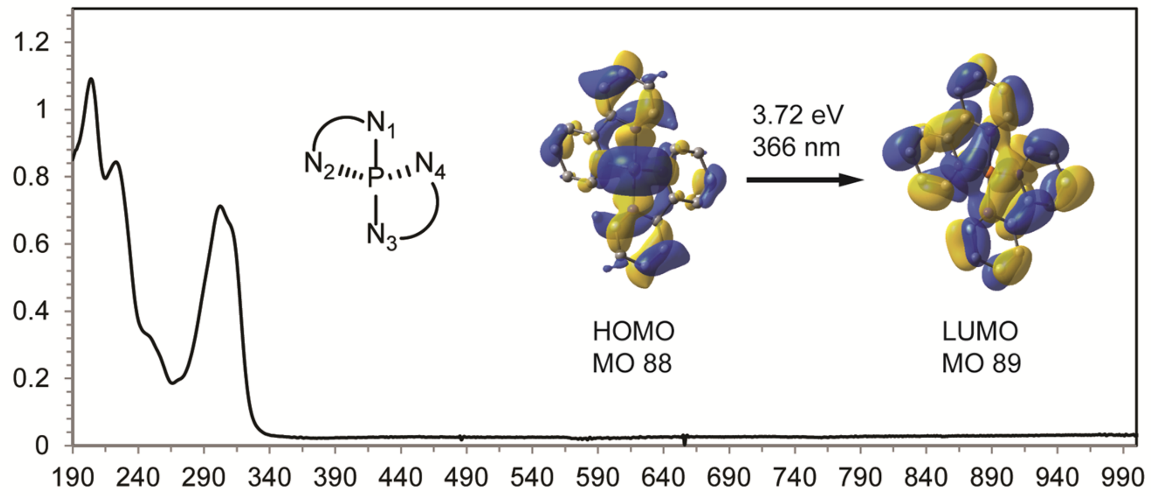

Figure S1. UV-Vis spectrum of [**6'P**][OTf]$_3$ in MeCN. Calculated (time-dependent DFT) energy for the HOMO-LUMO transition in model cation [**6P**]$^{3+}$ at the PBE0/def2-TZVP level in an acetonitrile field.

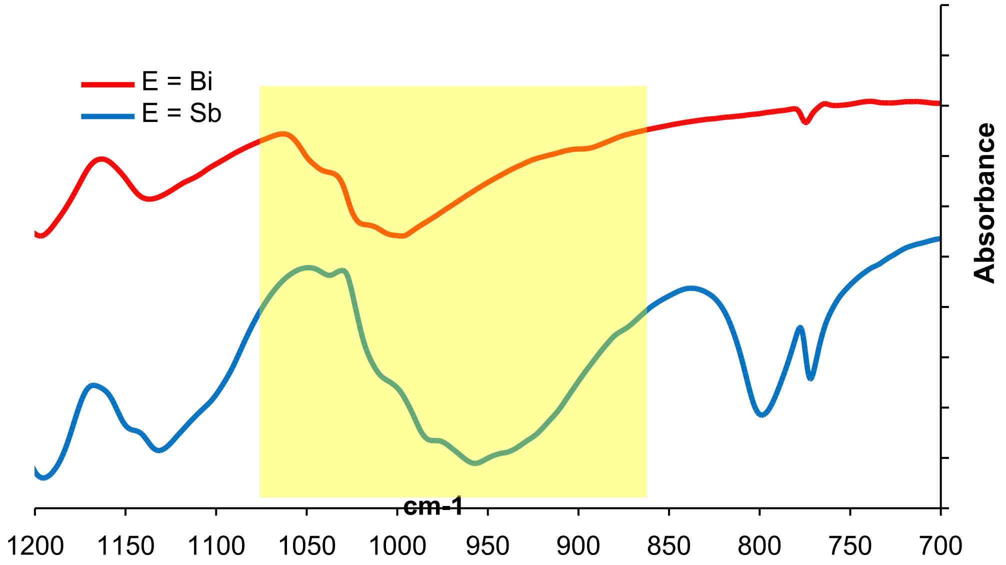

Figure S2. Selected regions of the infrared spectra of E(OTf)$_3$. The υ(SO_3) region is highlighted.

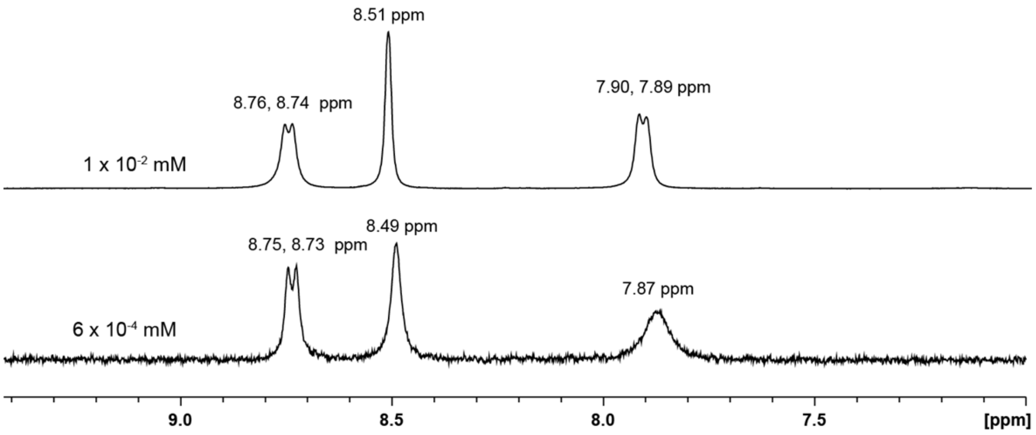

Figure S3. ^{1}H NMR spectra (CD_3CN, 238 K, 300.27 MHz) of [**6’Bi**][OTf]$_3$ at differing concentrations.

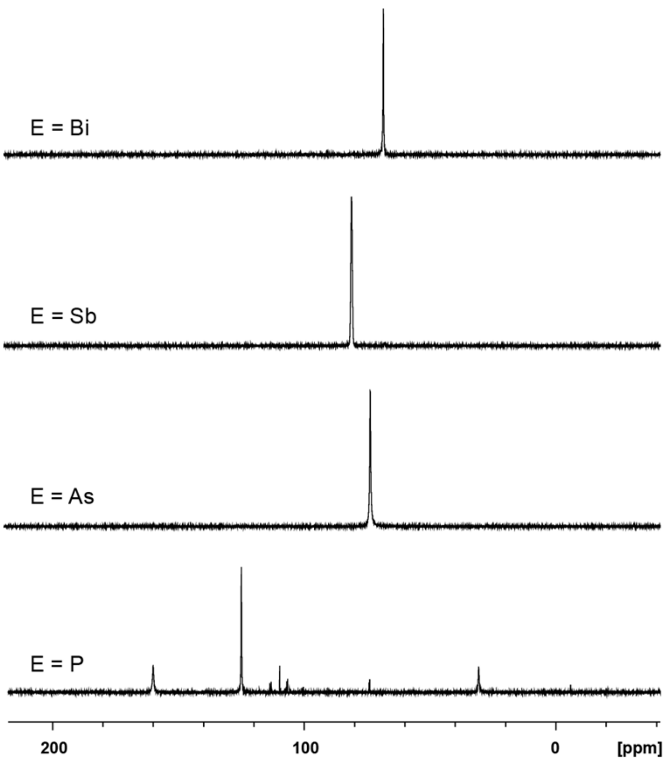

Figure S4. ^{31}P NMR spectra (CD_3CN, 298 K, 202.5 MHz) of solutions containing equimolar amounts of [**6’E**][OTf]$_3$ and Et_3PO.

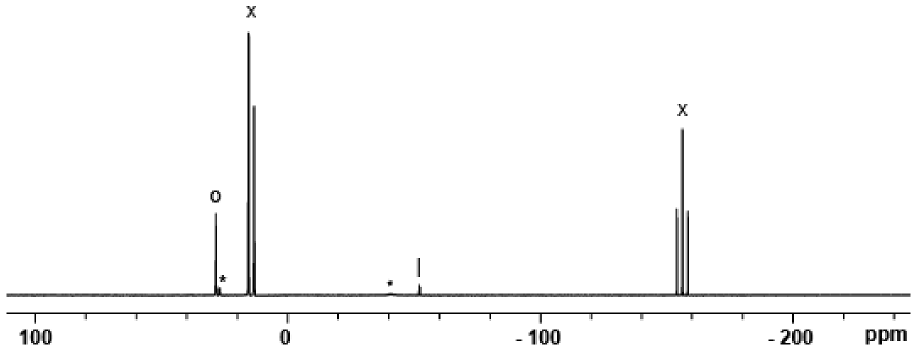

Figure S5. $^{31}P\{^{1}H\}$ NMR spectrum (CD_3CN, 298 K, 202.5 MHz) of the reaction mixture containing [**6'P**][OTf]$_3$ and PMe_3 in a 1:3 ratio. Symbols denote resonances due to $[(Me_3P)_2P]^{1+}$ (x), $[Me_3PPMe_3]^{2+}$ (o), PMe_3 (|), and unidentified species (*).

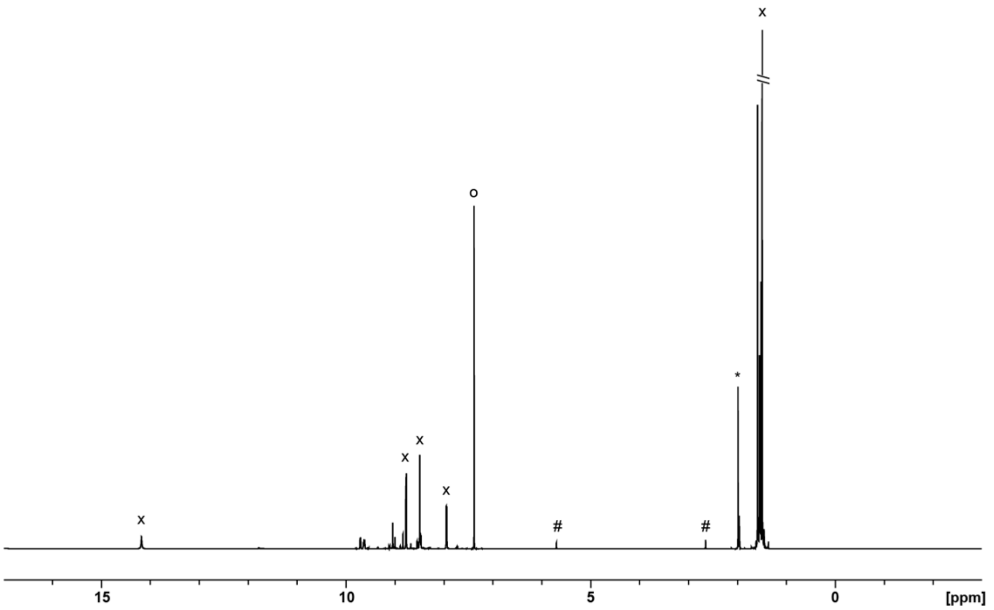

Figure S6. ^{1}H NMR spectrum (CD_3CN, 298 K, 500.27 MHz) of the 1:1 reaction mixture containing [**6'P**][OTf]$_3$ and 1,4-cyclohexadiene heated to 80 °C for 16 h in CD_3CN. Symbols denote signals due to [*tbbipy*-H]$^{1+}$ (x), benzene (o), 1,4-cyclohexadiene (#), and residual solvent (*).

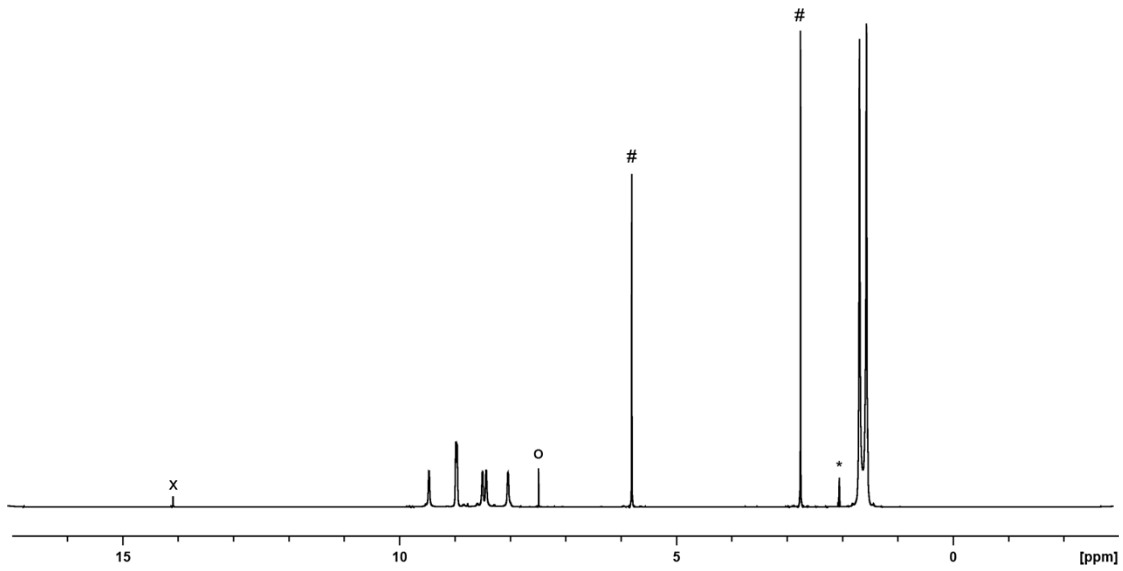

Figure S7. ^{1}H NMR spectrum (CD_3CN, 298 K, 500.27 MHz) of the 1:1 reaction mixture containing [**6’As**][OTf]$_3$ and 1,4-cyclohexadiene heated to 80 °C for 16 h in CD_3CN. Symbols denote signals due to [*tbbipy*-H]$^{1+}$ (x), benzene (o), 1,4-cyclohexadiene (#), and residual solvent (*).

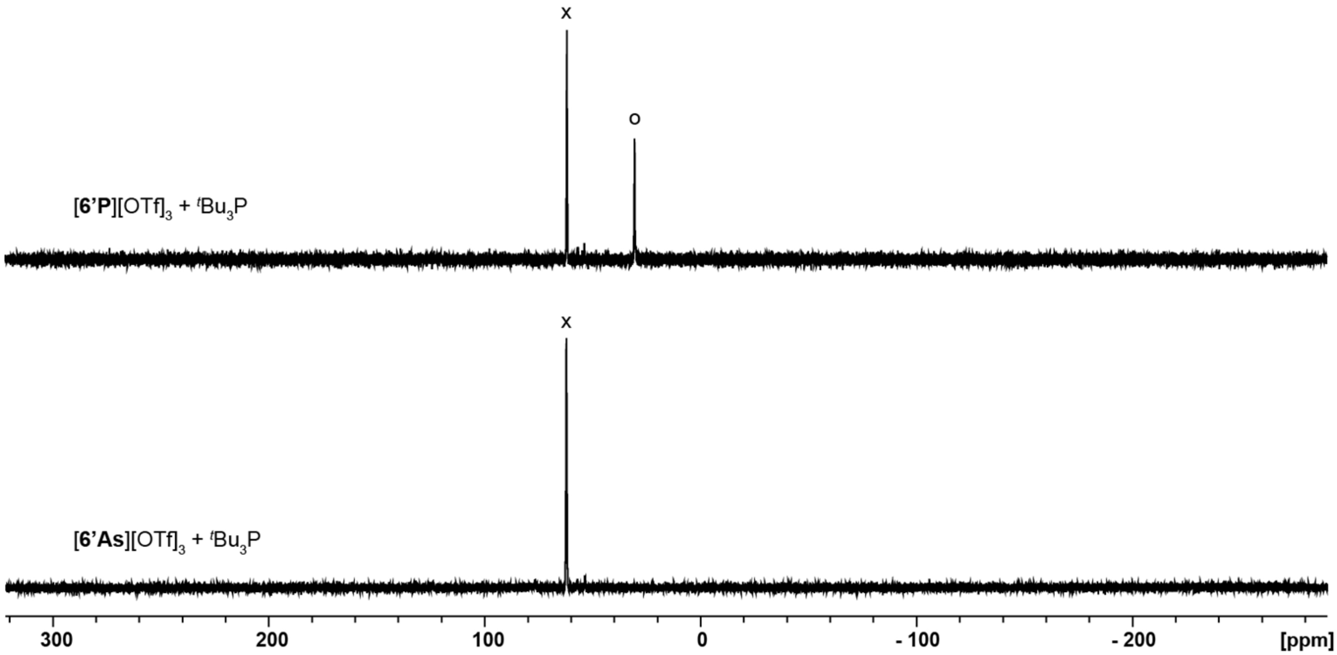

Figure S8. ^{31}P NMR spectrum (CD_3CN, 298 K, 145.85 MHz) of the 1:1 reaction mixture containing [**6’P**][OTf]$_3$ and $^{t}Bu_3P$ (top) and [**6’P**][OTf]$_3$ and $^{t}Bu_3P$ (bottom) after stirring for 1 h in CD_3CN. Symbols denote signals due to $^{t}Bu_3P$ (x), and [**6’P**][OTf]$_3$ (o).

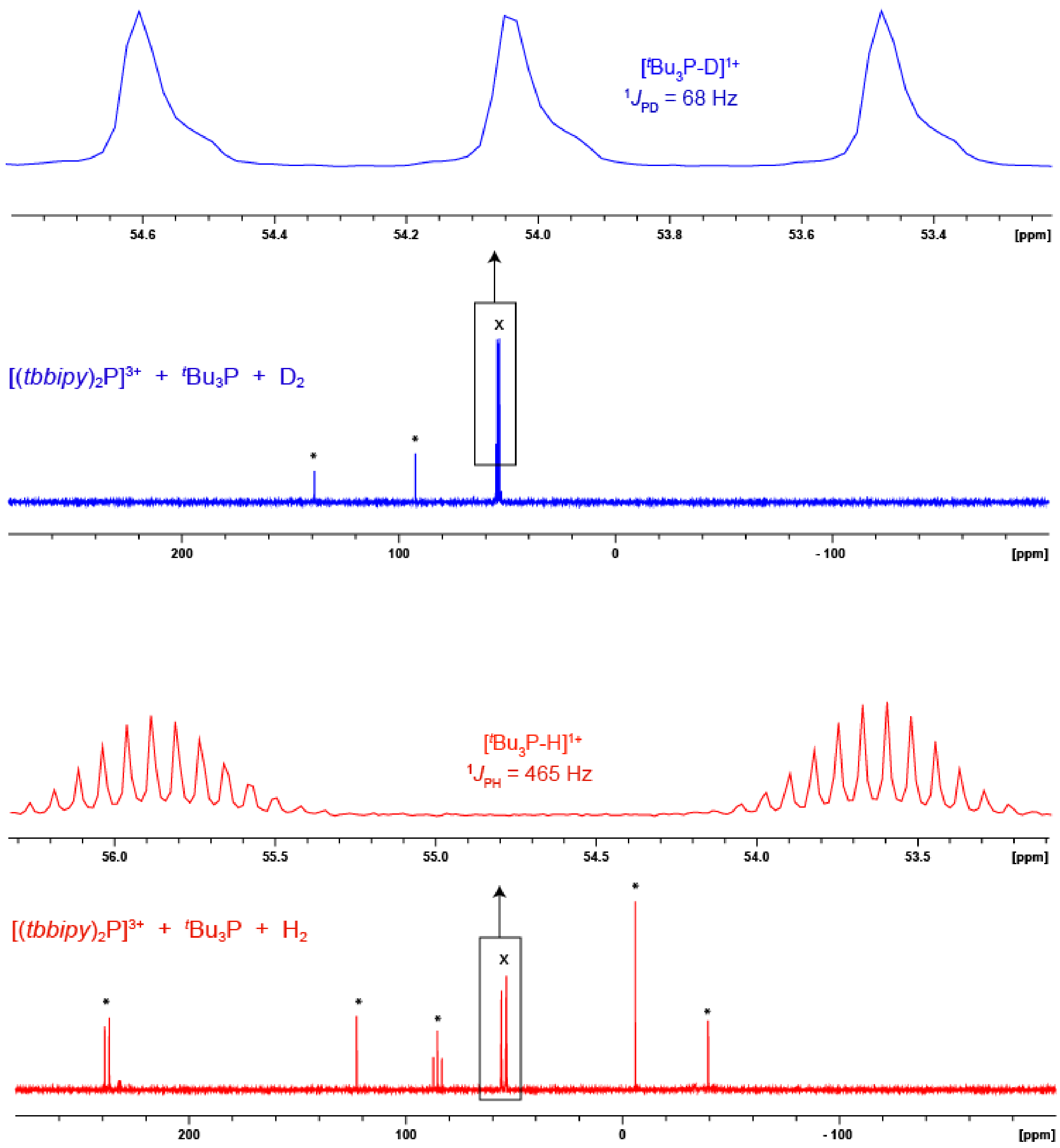

Figure S9. ^{31}P NMR spectra of reaction mixtures containing [**6'P**][OTf]$_3$, ^{t}Bu$_3$P, and H_2 (red) or D_2 (blue). Symbols denote signals due to [^{t}Bu$_3$P-H/D]$^{1+}$ (x) and unidentified products (*).

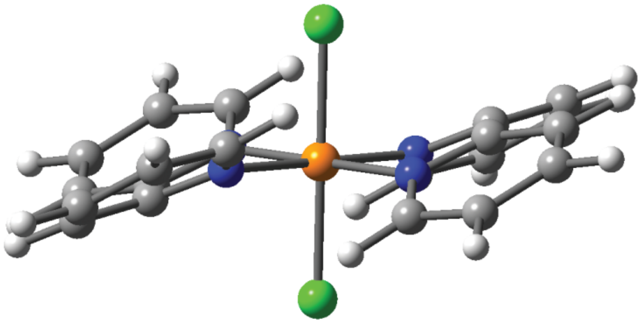

Figure S10. Gas-phase optimized (PBE0/def2-TZVP) structure of *trans*-$[(bipy)_2PCl_2]^{3+}$.

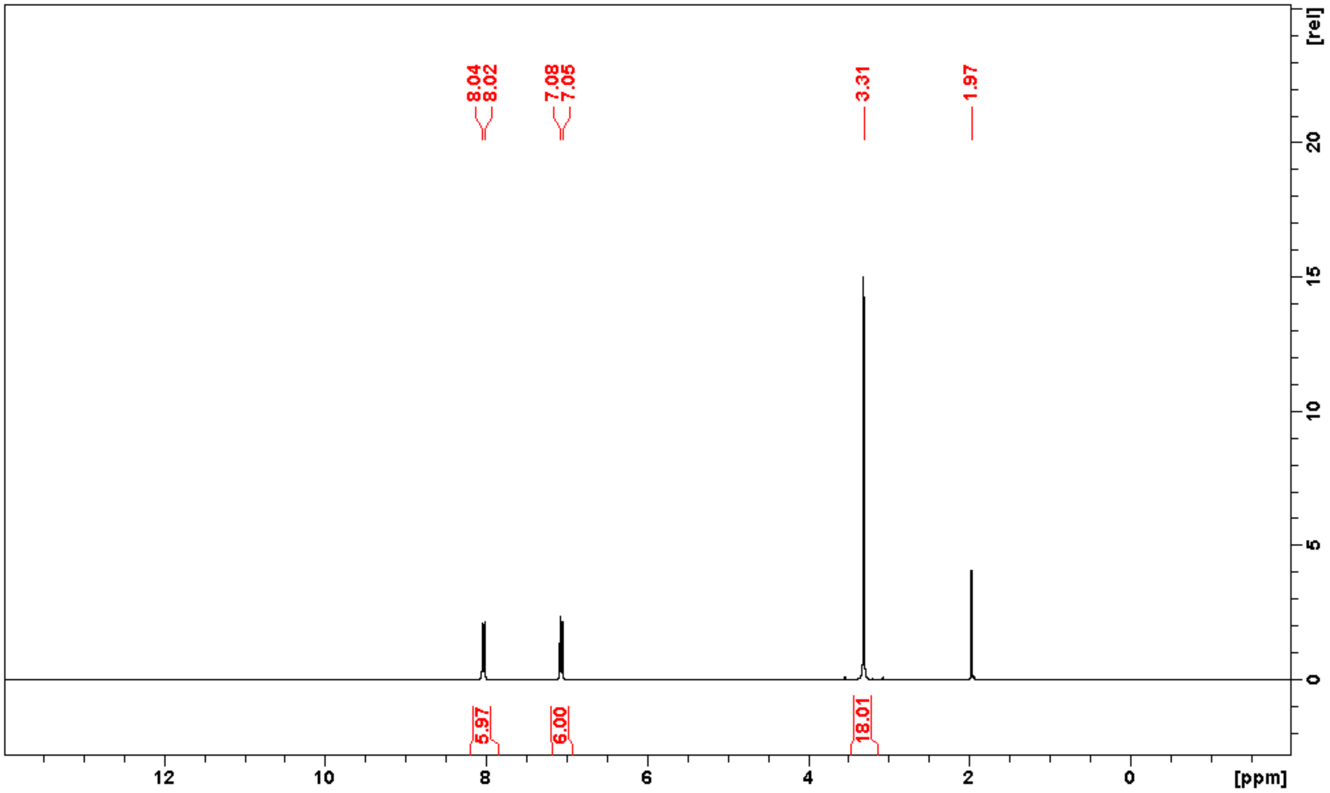

Figure S8. ^{1}H NMR spectrum of [**7P**][OTf]$_3$ in CD_3CN.

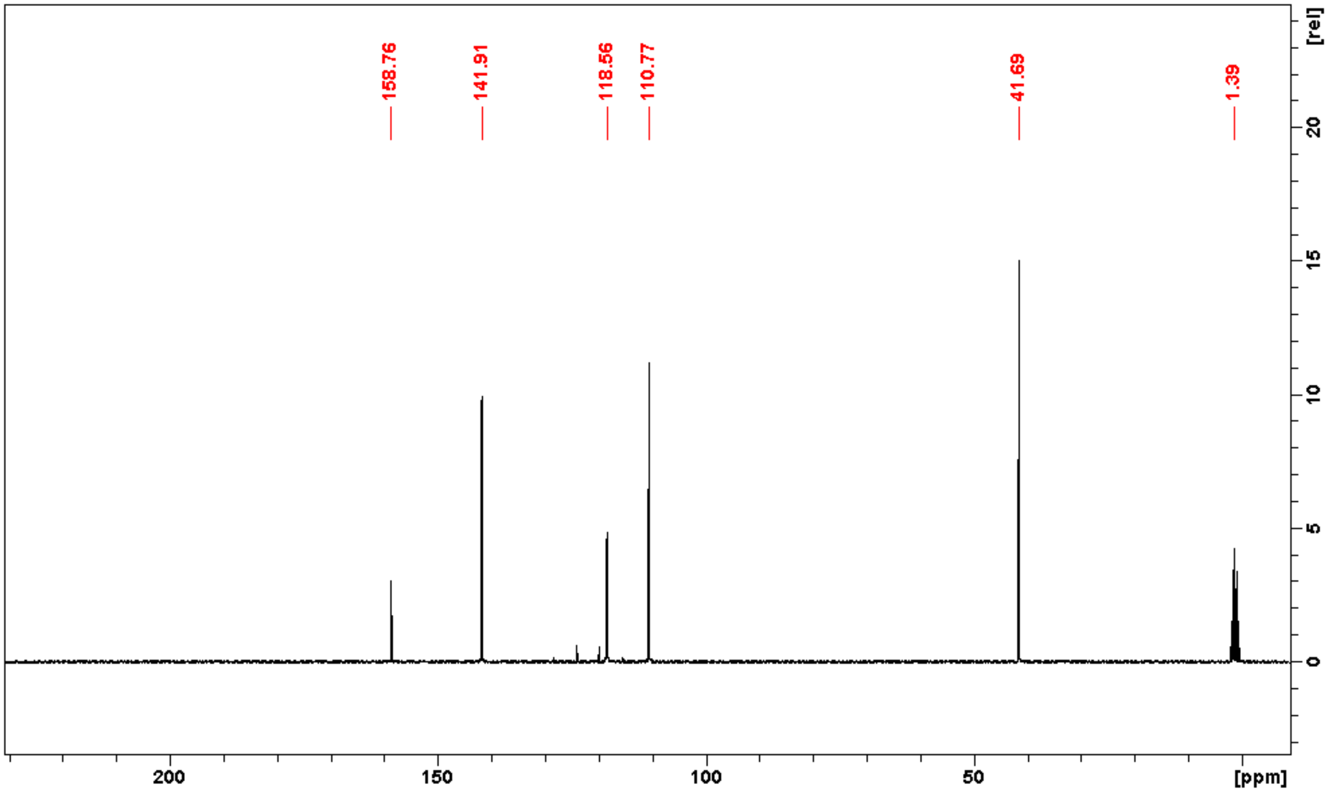

Figure S9. $^{13}C\{^{1}H\}$ NMR spectrum of [**7P**][OTf]$_3$ in CD_3CN.

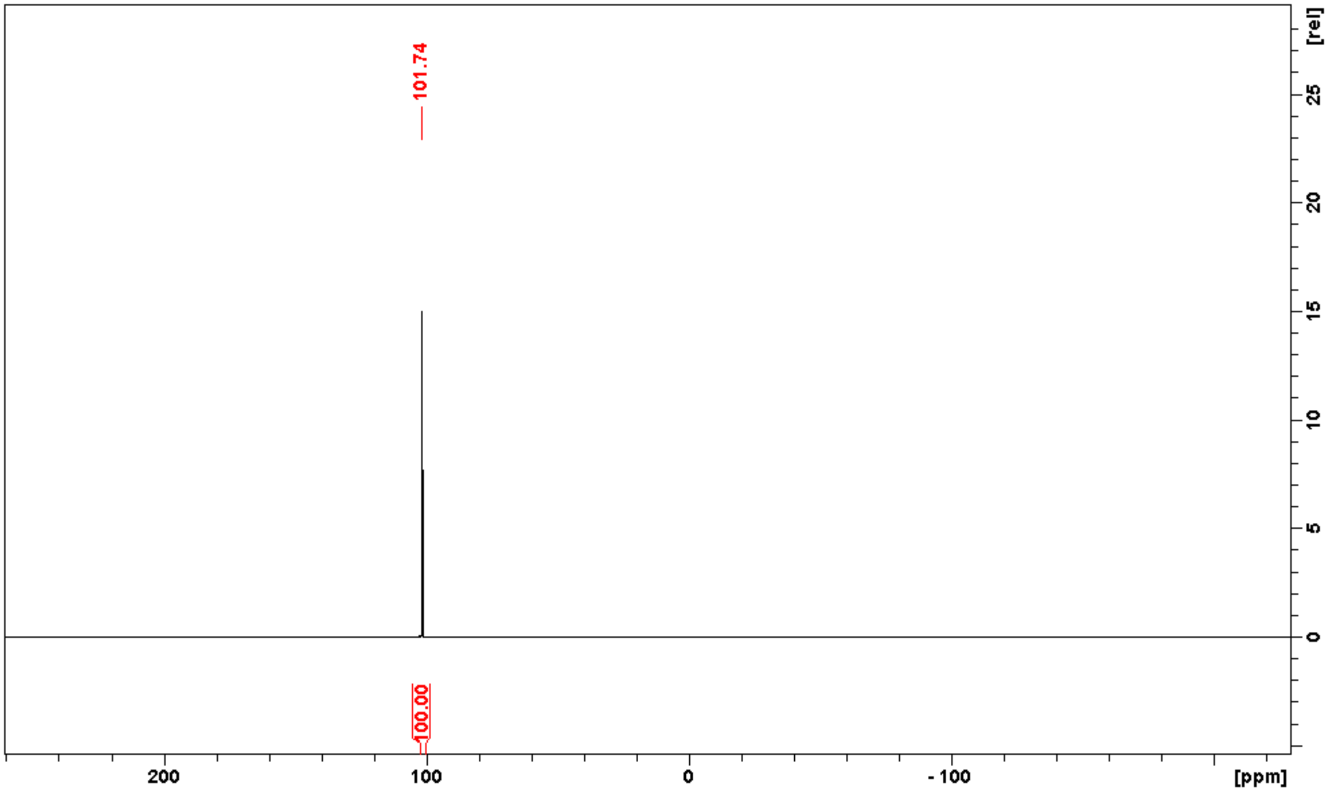

Figure S10. $^{31}P\{^{1}H\}$ NMR spectrum of [**7P**][OTf]$_3$ in CD_3CN.

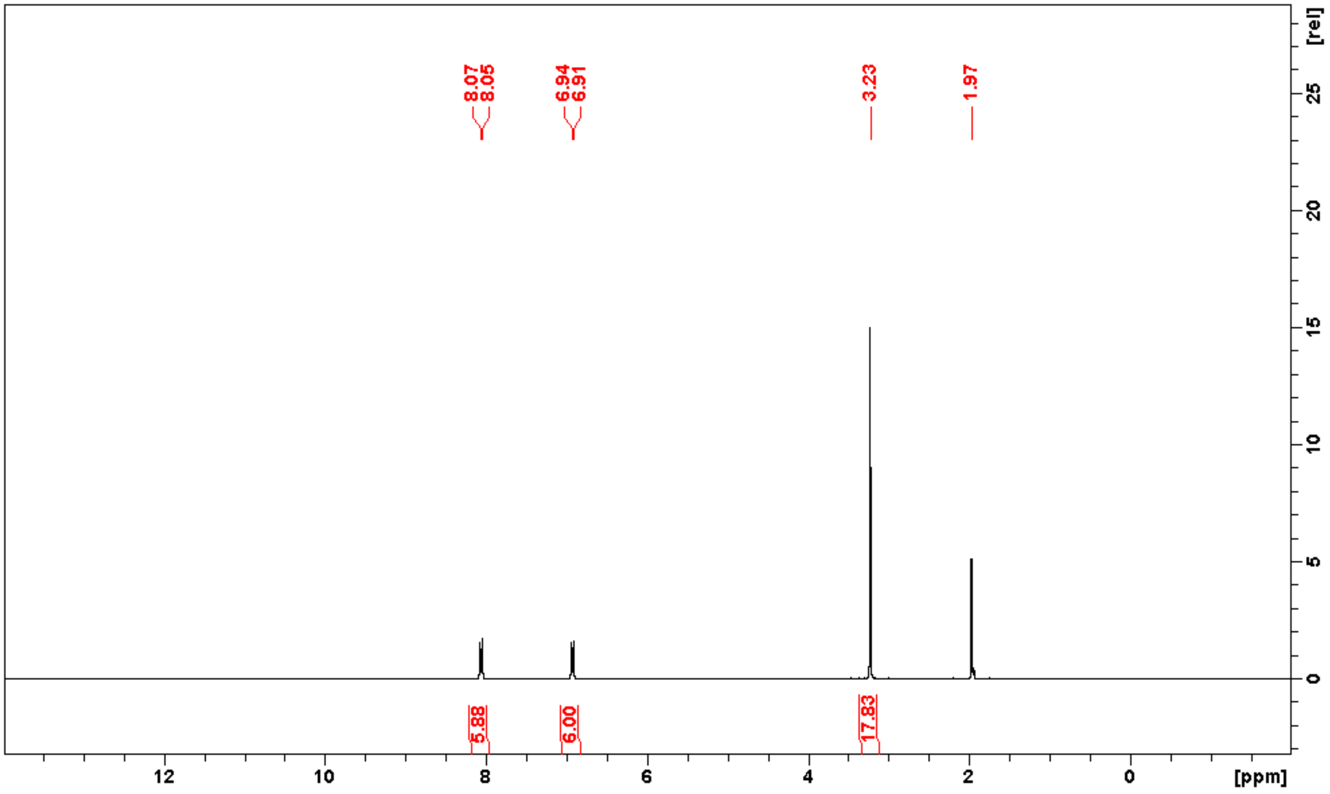

Figure S11. ^{1}H NMR spectrum of [**7As**][OTf]$_3$ in CD_3CN.

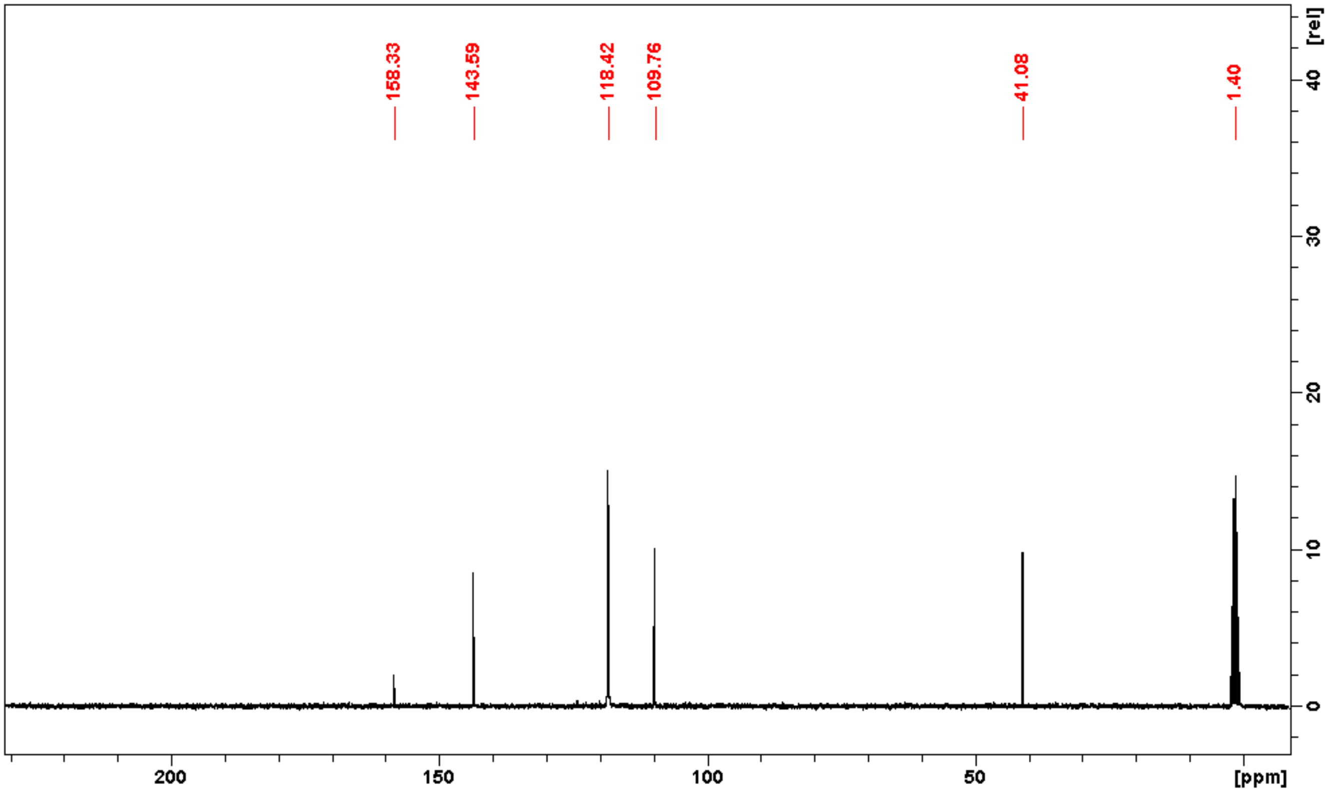

Figure S12. $^{13}C\{^{1}H\}$ NMR spectrum of [**7As**][OTf]$_3$ in CD_3CN.

Cartesian Coordinates of Optimized Geometries (PBE1PBE/def2-TZVP):

$[(bipy)_2P]^{3+}$

P 0.00000 0.00000 0.79635
N 1.80768 -0.73060 0.72276
N 0.69434 1.21821 -0.36766
N -0.69434 -1.21821 -0.36766
N -1.80768 0.73060 0.72276
C 2.19187 -1.76060 1.49178
H 1.41270 -2.40063 1.89296
C 3.52825 -1.98383 1.75338
H 3.82132 -2.83459 2.35702
C 4.47351 -1.09711 1.24763
H 5.52757 -1.24413 1.45695
C 4.06115 -0.00612 0.49345
H 4.78263 0.71333 0.12717
C 2.70860 0.14729 0.24665
C 2.05821 1.23211 -0.46946
C 2.69895 2.20206 -1.21388
H 3.77956 2.19901 -1.28157
C 1.95329 3.15623 -1.88831
H 2.44733 3.92099 -2.47827
C 0.56589 3.10507 -1.81376
H -0.05563 3.81524 -2.34588
C -0.03451 2.13433 -1.04979
H -1.10825 2.07059 -0.97296
C 0.03451 -2.13433 -1.04979
H 1.10825 -2.07059 -0.97296
C -0.56589 -3.10507 -1.81376
H 0.05563 -3.81524 -2.34588
C -1.95329 -3.15623 -1.88831
H -2.44733 -3.92098 -2.47828
C -2.69895 -2.20206 -1.21388
H -3.77956 -2.19901 -1.28157
C -2.05821 -1.23211 -0.46946
C -2.70860 -0.14729 0.24665
C -4.06115 0.00612 0.49345
H -4.78263 -0.71333 0.12717
C -4.47351 1.09711 1.24763
H -5.52757 1.24413 1.45695
C -3.52825 1.98383 1.75338
H -3.82132 2.83459 2.35702
C -2.19187 1.76060 1.49178
H -1.41270 2.40063 1.89296

$[(bipy)_2As]^{3+}$

As -0.00000 -0.00000 0.92446
N 1.97421 -0.68211 0.71364
N 0.71418 1.29727 -0.37683
N -0.71418 -1.29727 -0.37683
N -1.97421 0.68211 0.71364
C 2.47616 -1.67258 1.46341
H 1.76979 -2.32453 1.96787
C 3.84059 -1.85309 1.58340
H 4.22490 -2.66999 2.18254
C 4.69151 -0.96545 0.93725
H 5.76698 -1.07581 1.02456
C 4.16015 0.08209 0.19454
H 4.81731 0.79897 -0.28060
C 2.78406 0.19993 0.10258
C 2.06083 1.26673 -0.58677
C 2.65437 2.20558 -1.40975
H 3.72260 2.17209 -1.58044
C 1.87992 3.17726 -2.02491
H 2.34248 3.91599 -2.67077
C 0.50811 3.18510 -1.80953
H -0.13583 3.92058 -2.27635
C -0.04394 2.23602 -0.98145
H -1.10586 2.21120 -0.79048
C 0.04394 -2.23602 -0.98146
H 1.10586 -2.21120 -0.79048
C -0.50811 -3.18510 -1.80954
H 0.13584 -3.92057 -2.27637
C -1.87992 -3.17725 -2.02492
H -2.34247 -3.91598 -2.67079
C -2.65437 -2.20557 -1.40976
H -3.72259 -2.17208 -1.58046
C -2.06083 -1.26673 -0.58678
C -2.78406 -0.19993 0.10257
C -4.16015 -0.08209 0.19453
H -4.81731 -0.79897 -0.28062
C -4.69151 0.96544 0.93724
H -5.76699 1.07581 1.02455
C -3.84060 1.85308 1.58340
H -4.22490 2.66998 2.18254
C -2.47616 1.67258 1.46341
H -1.76979 2.32452 1.96788

$[(bipy)_2Sb]^{3+}$

Sb -0.00001 -0.00001 1.09174

N 2.12273 -0.61700 0.64889
N 0.74334 1.41750 -0.37012
N -0.74334 -1.41749 -0.37015
N -2.12273 0.61699 0.64888
C 2.73434 -1.59507 1.33388
H 2.11132 -2.23964 1.94687
C 4.10116 -1.77814 1.26806
H 4.56628 -2.58185 1.82618
C 4.84860 -0.90577 0.48916
H 5.92581 -1.01360 0.42320
C 4.21235 0.12441 -0.19164
H 4.79815 0.82594 -0.77046
C 2.83617 0.25102 -0.09302
C 2.05594 1.32889 -0.71542
C 2.59788 2.24009 -1.60542
H 3.63720 2.16216 -1.89498
C 1.80976 3.25121 -2.13382
H 2.23578 3.96747 -2.82806
C 0.47530 3.33167 -1.76348
H -0.17650 4.10635 -2.14849
C -0.02377 2.39898 -0.88293
H -1.05938 2.42582 -0.57572
C 0.02378 -2.39896 -0.88297
H 1.05938 -2.42581 -0.57575
C -0.47529 -3.33164 -1.76354
H 0.17652 -4.10632 -2.14855
C -1.80974 -3.25117 -2.13389
H -2.23576 -3.96743 -2.82815
C -2.59787 -2.24006 -1.60549
H -3.63718 -2.16213 -1.89505
C -2.05594 -1.32888 -0.71547
C -2.83617 -0.25102 -0.09306
C -4.21235 -0.12441 -0.19169
H -4.79814 -0.82593 -0.77052
C -4.84861 0.90576 0.48912
H -5.92582 1.01360 0.42315
C -4.10117 1.77812 1.26804
H -4.56629 2.58182 1.82618
C -2.73435 1.59504 1.33388
H -2.11134 2.23961 1.94689

[(*bipy*)$_2$Sb]$^{3+}$

Bi -0.00001 -0.00001 1.03034
N 2.20813 -0.63558 0.48906
N 0.81493 1.46183 -0.49997
N -0.81492 -1.46183 -0.50000
N -2.20814 0.63558 0.48905
C 2.84370 -1.63074 1.12442
H 2.24442 -2.29208 1.74334
C 4.20664 -1.81946 1.00676
H 4.68738 -2.63716 1.53010
C 4.92757 -0.93424 0.21920
H 6.00105 -1.04312 0.10995
C 4.26821 0.10808 -0.41858
H 4.83598 0.81520 -1.00789
C 2.89607 0.24069 -0.26779
C 2.11491 1.33589 -0.86937
C 2.65674 2.23122 -1.77798
H 3.68554 2.12842 -2.09489
C 1.88189 3.25922 -2.29287
H 2.31034 3.96088 -3.00027
C 0.55867 3.37541 -1.89450
H -0.08222 4.16423 -2.26878
C 0.05913 2.45571 -0.99956
H -0.97042 2.50423 -0.67130
C -0.05912 -2.45570 -0.99959
H 0.97043 -2.50422 -0.67132
C -0.55865 -3.37539 -1.89454
H 0.08225 -4.16420 -2.26884
C -1.88186 -3.25920 -2.29293
H -2.31031 -3.96084 -3.00035
C -2.65672 -2.23120 -1.77803
H -3.68552 -2.12839 -2.09496
C -2.11490 -1.33588 -0.86941
C -2.89607 -0.24068 -0.26783
C -4.26820 -0.10807 -0.41863
H -4.83597 -0.81518 -1.00796
C -4.92757 0.93424 0.21916
H -6.00106 1.04312 0.10989
C -4.20665 1.81945 1.00673
H -4.68740 2.63714 1.53008
C -2.84371 1.63073 1.12441
H -2.24444 2.29206 1.74334

[(*bipy*)$_2$Bi]$^{3+}$

Bi -0.00001 -0.00001 1.03034
N 2.20813 -0.63558 0.48906
N 0.81493 1.46183 -0.49997
N -0.81492 -1.46183 -0.50000
N -2.20814 0.63558 0.48905
C 2.84370 -1.63074 1.12442
H 2.24442 -2.29208 1.74334
C 4.20664 -1.81946 1.00676
H 4.68738 -2.63716 1.53010
C 4.92757 -0.93424 0.21920
H 6.00105 -1.04312 0.10995
C 4.26821 0.10808 -0.41858
H 4.83598 0.81520 -1.00789
C 2.89607 0.24069 -0.26779
C 2.11491 1.33589 -0.86937
C 2.65674 2.23122 -1.77798
H 3.68554 2.12842 -2.09489
C 1.88189 3.25922 -2.29287
H 2.31034 3.96088 -3.00027
C 0.55867 3.37541 -1.89450
H -0.08222 4.16423 -2.26878
C 0.05913 2.45571 -0.99956
H -0.97042 2.50423 -0.67130
C -0.05912 -2.45570 -0.99959
H 0.97043 -2.50422 -0.67132
C -0.55865 -3.37539 -1.89454
H 0.08225 -4.16420 -2.26884
C -1.88186 -3.25920 -2.29293
H -2.31031 -3.96084 -3.00035
C -2.65672 -2.23120 -1.77803
H -3.68552 -2.12839 -2.09496
C -2.11490 -1.33588 -0.86941
C -2.89607 -0.24068 -0.26783
C -4.26820 -0.10807 -0.41863
H -4.83597 -0.81518 -1.00796
C -4.92757 0.93424 0.21916
H -6.00106 1.04312 0.10989
C -4.20665 1.81945 1.00673
H -4.68740 2.63714 1.53008
C -2.84371 1.63073 1.12441
H -2.24444 2.29206 1.74334

[(*bipy*)$_2$P(OPMe$_3$)]$^{3+}$

N -1.44465 -1.14618 -0.76114
N -0.23061 -0.51102 1.33633
N 1.48460 -1.41230 -0.50397
N 1.78548 1.05427 0.16056
C -1.90359 -1.37418 -1.98968
H -1.32041 -0.97010 -2.81204
C -3.06235 -2.10520 -2.18152
H -3.42024 -2.30269 -3.18461
C -3.74548 -2.57651 -1.06603
H -4.65997 -3.14642 -1.18809
C -3.25422 -2.32226 0.20903
H -3.78027 -2.68788 1.08160
C -2.07795 -1.59975 0.32055
C -1.34708 -1.25805 1.53683
C -1.70639 -1.68157 2.80207
H -2.59763 -2.28143 2.93269
C -0.91614 -1.35176 3.89096
H -1.18704 -1.68013 4.88810
C 0.23098 -0.60696 3.67294
H 0.89282 -0.33237 4.48482
C 0.54034 -0.20382 2.39120
H 1.42805 0.37588 2.20215
C 1.21990 -2.69947 -0.76001
H 0.19669 -3.02823 -0.63941
C 2.20312 -3.58600 -1.14978
H 1.94880 -4.62244 -1.33351
C 3.50412 -3.12510 -1.29108
H 4.29762 -3.79566 -1.60032
C 3.77932 -1.79531 -1.02084
H 4.79023 -1.42108 -1.11205
C 2.74976 -0.95786 -0.62020
C 2.91325 0.45178 -0.27769
C 4.10333 1.15749 -0.37453
H 4.99949 0.67407 -0.73982
C 4.13818 2.48752 0.00670
H 5.06293 3.04956 -0.05726
C 2.97744 3.08676 0.48036
H 2.97173 4.11780 0.81164
C 1.81865 2.34320 0.53365
H 0.88930 2.75507 0.90200
O -1.00263 1.50590 0.13393
P -1.86752 2.60481 -0.57430
C -1.83698 4.08000 0.42629
H -2.17613 3.85428 1.43915
H -0.83424 4.50901 0.46105
H -2.51036 4.82265 -0.01089
C -1.24587 2.99175 -2.20348
H -1.28723 2.11877 -2.85670

H -1.86181 3.78149 -2.64226
H -0.21638 3.35015 -2.14469
C -3.55947 2.05527 -0.71183
H -3.62892 1.17071 -1.34695
H -3.96080 1.83256 0.27870
H -4.16255 2.84934 -1.16029
P 0.12395 0.10230 -0.43173

$[(bipy)_2As(OPMe_3)]^{3+}$

N 1.79888 1.25788 0.03522
N 1.69390 -1.30855 -0.56101
N -0.22942 -0.48089 1.33116
N -1.42263 -1.42147 -0.76302
C 1.73990 2.57048 0.29690
H 0.75517 2.96773 0.50574
C 2.87000 3.36067 0.31895
H 2.78850 4.41553 0.54949
C 4.09632 2.77363 0.03987
H 5.00413 3.36630 0.03835
C 4.15156 1.41792 -0.23980
H 5.10173 0.95408 -0.46693
C 2.98323 0.66987 -0.23254
C 2.92896 -0.76952 -0.51422
C 4.05349 -1.55377 -0.72309
H 5.04372 -1.12121 -0.67937
C 3.90343 -2.90615 -0.98334
H 4.77484 -3.52990 -1.14669
C 2.62775 -3.44645 -1.03024
H 2.46545 -4.49852 -1.22846
C 1.54791 -2.61296 -0.81797
H 0.53749 -2.99787 -0.85277
C 0.44655 0.03950 2.36543
H 1.19527 0.78137 2.13951
C 0.21056 -0.34576 3.66782
H 0.78761 0.10342 4.46636
C -0.76138 -1.29986 3.91959
H -0.97102 -1.62781 4.93152
C -1.46414 -1.83097 2.85093
H -2.22615 -2.57977 3.02069
C -1.18966 -1.41030 1.56120
C -1.88786 -1.92737 0.38132
C -2.92376 -2.84858 0.38150
H -3.31292 -3.26283 1.30224
C -3.46249 -3.23803 -0.83837
H -4.27088 -3.96021 -0.86556
C -2.96296 -2.70192 -2.01811
H -3.36118 -2.99183 -2.98276
C -1.93311 -1.78133 -1.94002
H -1.49874 -1.32751 -2.82594
O -1.18947 1.60640 0.06913
P -2.28637 2.55961 -0.48955
C -2.04786 2.90313 -2.22789
H -1.07113 3.36107 -2.39635
H -2.12715 1.98935 -2.81984
H -2.81764 3.59963 -2.57035
C -3.90468 1.83732 -0.26951
H -4.08570 1.64609 0.78989
H -4.66932 2.52795 -0.63422
H -3.98641 0.90458 -0.83042
C -2.23393 4.10113 0.40757
H -2.35301 3.91665 1.47686
H -1.29126 4.62119 0.22794
H -3.04980 4.74617 0.07045
As 0.08599 0.13388 -0.57156

$[(bipy)_2Sb(OPMe_3)]^{3+}$

N 1.69060 1.51051 -0.06619
N 1.92647 -1.10997 -0.59007
N -0.26353 -0.48472 1.33638
N -1.27447 -1.80057 -0.72403
C 1.49455 2.82319 0.12085
H 0.46234 3.14763 0.16104
C 2.54084 3.70928 0.26787
H 2.34267 4.76210 0.42471
C 3.83583 3.21688 0.20258
H 4.68582 3.88210 0.30437
C 4.03610 1.86143 -0.00196
H 5.04389 1.47584 -0.06764
C 2.94379 1.01527 -0.13430
C 3.07143 -0.43245 -0.37449
C 4.29469 -1.08708 -0.39944
H 5.21445 -0.54884 -0.21774
C 4.33962 -2.44714 -0.65990
H 5.29118 -2.96584 -0.68305
C 3.15619 -3.12829 -0.89442
H 3.14544 -4.18934 -1.10974
C 1.97148 -2.42090 -0.85178
H 1.02680 -2.91680 -1.03561
C 0.24048 0.24237 2.34253
H 0.77048 1.14425 2.07549
C 0.10163 -0.12340 3.66484
H 0.53088 0.49831 4.44040
C -0.58871 -1.28647 3.96314
H -0.71438 -1.61133 4.98979

C -1.12560 -2.02874 2.92472
H -1.67624 -2.93396 3.14031
C -0.95953 -1.61162 1.61328
C -1.53439 -2.34174 0.47212
C -2.30105 -3.49336 0.57324
H -2.52118 -3.94174 1.53253
C -2.79535 -4.07484 -0.58679
H -3.39593 -4.97553 -0.52607
C -2.51870 -3.49770 -1.81797
H -2.89000 -3.92593 -2.74083
C -1.74876 -2.34980 -1.84458
H -1.49845 -1.85688 -2.77967
O -1.43199 1.62499 -0.04917
P -2.71887 2.43589 -0.37181
C -3.28457 2.13315 -2.04036
H -2.51724 2.41844 -2.76310
H -3.54369 1.08078 -2.17264
H -4.17866 2.73059 -2.23681
C -4.01984 1.98524 0.76326
H -3.70554 2.18644 1.78908
H -4.91583 2.57398 0.55036
H -4.26595 0.92714 0.65837
C -2.39585 4.18301 -0.19855
H -2.05495 4.40551 0.81459
H -1.64736 4.50843 -0.92388
H -3.31615 4.74378 -0.38239
Sb -0.01005 0.15604 -0.77622

$[(bipy)_2Bi(OPMe_3)]^{3+}$

Bi -0.05627 0.10876 -0.76082
N 1.39538 1.84464 0.00532
N 2.22372 -0.67492 -0.54141
N -0.25413 -0.59488 1.42405
N -0.80080 -2.20820 -0.65837
C 0.92396 3.08462 0.19005
H -0.15406 3.19190 0.16284
C 1.75434 4.16443 0.40963
H 1.33556 5.15180 0.55771
C 3.12330 3.94681 0.42564
H 3.81195 4.76849 0.58662
C 3.60872 2.66474 0.22437
H 4.67642 2.49665 0.21990
C 2.72378 1.61548 0.01395
C 3.17222 0.23017 -0.23192
C 4.50772 -0.14357 -0.16924
H 5.27270 0.57427 0.09121
C 4.86934 -1.45298 -0.44280
H 5.91092 -1.75000 -0.39890
C 3.88541 -2.36917 -0.77549
H 4.12334 -3.40008 -1.00532
C 2.57446 -1.93682 -0.81081
H 1.77593 -2.62322 -1.06555
C 0.00265 0.25836 2.42384
H 0.28778 1.26351 2.14604
C -0.08535 -0.10863 3.74994
H 0.13436 0.61487 4.52485
C -0.45416 -1.40965 4.05092
H -0.52894 -1.74147 5.08032
C -0.73363 -2.28561 3.01543
H -1.02988 -3.30038 3.24106
C -0.63501 -1.86302 1.69738
C -0.95318 -2.74747 0.55950
C -1.40170 -4.05351 0.70070
H -1.53198 -4.50149 1.67606
C -1.69458 -4.79571 -0.43485
H -2.04636 -5.81665 -0.33807
C -1.53821 -4.22135 -1.68696
H -1.76076 -4.76829 -2.59479
C -1.08694 -2.91625 -1.75413
H -0.94841 -2.42446 -2.71269
O -1.81374 1.41556 -0.13914
P -3.25149 1.96813 -0.30183
C -3.91724 1.59201 -1.91792
H -3.29346 2.03301 -2.69822
H -3.98159 0.51191 -2.06417
H -4.92377 2.00839 -2.00803
C -4.33506 1.26416 0.93114
H -3.96821 1.50300 1.93101
H -5.34061 1.67774 0.82072
H -4.39107 0.18063 0.81235
C -3.25131 3.74312 -0.10174
H -2.87751 4.01205 0.88813
H -2.63466 4.21219 -0.87108
H -4.27107 4.12423 -0.19982
